# Cognition-Oriented Treatments for Older Adults: a Systematic Overview of Systematic Reviews

**DOI:** 10.1007/s11065-020-09434-8

**Published:** 2020-04-07

**Authors:** Hanna Malmberg Gavelin, Amit Lampit, Harry Hallock, Julieta Sabatés, Alex Bahar-Fuchs

**Affiliations:** 1 Department of Psychology, Umeå University, Umeå, Sweden; 2 Academic Unit for Psychiatry of Old Age, University of Melbourne, Melbourne, Australia; 3 Department of Neurology, Charité–Universitätsmedizin Berlin, Berlin, Germany; 4 Berlin School of Mind and Brain, Humboldt-Universität zu Berlin, Berlin, Germany

**Keywords:** Cognitive training, Cognitive stimulation, Cognitive rehabilitation, Older adults, Systematic review, Overview

## Abstract

Cognition-oriented treatments – commonly categorized as cognitive training, cognitive rehabilitation and cognitive stimulation – are promising approaches for the prevention of cognitive and functional decline in older adults. We conducted a systematic overview of meta-analyses investigating the efficacy of cognition-oriented treatments on cognitive and non-cognitive outcomes in older adults with or without cognitive impairment. Review quality was assessed by A Measurement Tool to Assess Systematic Reviews 2 (AMSTAR). We identified 51 eligible reviews, 46 of which were included in the quantitative synthesis. The confidence ratings were “moderate” for 9 (20%), “low” for 13 (28%) and “critically low” for 24 (52%) of the 46 reviews. While most reviews provided pooled effect estimates for objective cognition, non-cognitive outcomes of potential relevance were more sparsely reported. The mean effect estimate on cognition was small for cognitive training in healthy older adults (mean Hedges’ *g* = 0.32, range 0.13–0.64, 19 reviews), mild cognitive impairment (mean Hedges’ *g* = 0.40, range 0.32–0.60, five reviews), and dementia (mean Hedges’ *g* = 0.38, range 0.09–1.16, seven reviews), and small for cognitive stimulation in dementia (mean Hedges’ *g* = 0.36, range 0.26–0.44, five reviews). Meta-regression revealed that higher AMSTAR score was associated with larger effect estimates for cognitive outcomes. The available evidence supports the efficacy of cognition-oriented treatments improving cognitive performance in older adults. The extent to which such effects are of clinical value remains unclear, due to the scarcity of high-quality evidence and heterogeneity in reported findings. An important avenue for future trials is to include relevant non-cognitive outcomes in a more consistent way and, for meta-analyses in the field, there is a need for better adherence to methodological standards. PROSPERO registration number: CRD42018084490.

**Electronic supplementary material:**

The online version of this article (10.1007/s11065-020-09434-8) contains supplementary material, which is available to authorized users.

## Introduction

As a larger proportion of the population reaches more advanced age, more people will be affected by cognitive aging. Some cognitive decline occurs in what is usually considered normal ageing (Deary et al., 2009; Nyberg, Lovden, Riklund, Lindenberger, & Backman, 2012), and it is a prominent feature in several predominantly age-related pathological conditions, including neurodegenerative and neurovascular diseases (Aarsland et al., 2017; Cumming, Marshall, & Lazar, 2013; Weintraub, Wicklund, & Salmon, 2012). Given the functional consequences of cognitive impairment in aging, and the subsequent personal, societal and financial costs, development of effective interventions that could maintain levels of cognitive functioning and delay cognitive and functional decline is a key priority in the field.

Cognition-oriented treatments is an umbrella term referring to several non-pharmacological treatment approaches which apply a range of techniques to engage thinking and cognition with various degrees of breadth and specificity. Unlike treatments that are primarily oriented towards outcomes that are behavioural (e.g. wandering), emotional (e.g. anxiety), or physical (e.g. sedentary lifestyle), in cognition-oriented treatments, the goals include improving or maintaining cognitive processes or addressing the impact of impairment in cognitive processes on associated functional ability in daily life (Bahar-Fuchs, Martyr, Goh, Sabates, & Clare, 2019). Cognitive training involves repeated practice on a set of structured and standardized tasks, designed to target one or several cognitive abilities (Bahar-Fuchs, Clare, & Woods, 2019; Clare & Woods, 2004). Cognitive stimulation consists of non-specific engagement in a variety of activities aimed at general enhancement of cognitive and social functioning, usually carried out in group settings at clinics or residential care facilities (Woods, Aguirre, Spector, & Orrell, 2012). Cognitive rehabilitation is an individualized approach with a functional emphasis, aimed at achieving or maintaining optimal levels of functioning in everyday life (Clare & Woods, 2004; Kudlicka, Martyr, Bahar-Fuchs, Woods, & Clare, 2019). Core elements of cognitive rehabilitation include identifying personally-relevant goals and devising strategies for goal achievement in collaboration with the patients and their families (Bahar-Fuchs et al., 2019). Thus, the focus of cognitive training is on restoring specific cognitive abilities, whereas cognitive stimulation is directed towards improving overall orientation and global cognitive status, and cognitive rehabilitation is focused on producing functional change in the everyday context (Bahar-Fuchs et al., 2019).

Given the potential of cognition-oriented treatments to maintain cognitive health in old age, the field has received widespread attention in recent years. Yet, the extent to which the three intervention approaches are efficacious on cognitive and non-cognitive outcomes in older adults, and whether such effects translate into prevention of further cognitive decline, remains hotly debated, as reflected in the highly publicised consensus letter (Stanford Center on Longevity, 2014) and counter letter (Cognitive Training Data, 2014) from the scientific community regarding cognitive training. Indeed, numerous systematic reviews and meta-analyses on these topics published in recent years have not produced clear and coherent evidence. Some of the contrasting findings from previous reviews are likely due to conceptual issues related to the various interventions, as well as differing methodological approaches by the review authors. Moreover, most meta-analyses have focused on one particular type of cognition-oriented treatment, group of older adults or outcome category. Therefore, there is a need to synthesize the available body of evidence, while also taking into account the methodological quality of published reviews.

In recent years, systematic overviews have emerged as a new form of evidence synthesis, allowing researchers to systematically identify and synthesize the available evidence from multiple systematic reviews on a given topic, resolve discrepancies and identify gaps in the literature (H. Cooper & Koenka, 2012; McKenzie & Brennan, 2017). Thus, a systematic overview can address broader research synthesis questions than a single systematic review (H. Cooper & Koenka, 2012). Such an approach to the efficacy of the three main types of cognition-oriented treatments on cognitive and non-cognitive outcomes for older adults on the spectrum from cognitive health to dementia could be of value for both research and practice and provide future directions for the field. This overview provides researchers and clinicians alike with a comprehensive and accessible summary of the available evidence, while also using a rigorous quality assessment process to evaluate the methodological quality of published meta-analyses, and explore its relation to review outcomes. This approach could therefore address some of the previous discrepancies in the field and identify areas in which further high-quality research is needed.

The aim of the present overview is to synthesize systematic reviews with meta-analysis of cognition-oriented treatments in older adults (aged >50 years). We sought to (1) provide an overview of the available evidence on the efficacy of the three main types of cognition-oriented treatments for older adults with or without cognitive decline on cognitive and non-cognitive outcomes, (2) explore the outcomes of reviews in relation to their methodological quality, (3) evaluate the strength and quality of the evidence for cognition-oriented treatments in older adults and, (4) suggest recommendations for further research and research synthesis in the field.

## Methods

### Protocol and Registration

This review follows the Preferred Reporting Items of Systematic Reviews and Meta-analyses (PRISMA) guidelines (Liberati et al., 2009) and the study protocol was prospectively registered with PROSPERO (CRD42018084490).

### Search Strategy and Study Selection

An electronic database search of MEDLINE, EMBASE, PsychINFO and Cochrane Database of Systematic Reviews was conducted from inception to April 2019 to identify systematic reviews with meta-analysis examining the effects of cognition-oriented treatments on cognitive or non-cognitive outcomes for older adults with or without cognitive decline (see Supplementary Material 1 for the full search strategy). No restrictions on language or publication type were applied. Initial screening of titles and abstracts was conducted by one reviewer (HMG) and full-text screening of potentially relevant papers was conducted independently by two reviewers (HMG and ABF, AL or JS). Disagreements were resolved by consensus and involvement of a third reviewer if necessary. The electronic search was complemented by hand-searching the references of retrieved articles.

### Eligibility Criteria

**Type of studies.** Systematic reviews using meta-analytic methods including randomized or non-randomized controlled trials were included. Reviews that included non-controlled trials were included if the results from controlled trials were reported separately. Overviews including a combination of reviews and primary trials were excluded.

**Types of participants.** Reviews were included if they focused on older adults (mean age > 50 years), with or without cognitive decline. This included cognitively unimpaired older adults (hereby referred to as “healthy older adults”), as well as conditions that are associated with cognitive impairment in older adults, such as mild cognitive impairment (MCI), dementia (irrespective of aetiology), Parkinson’s disease (PD) and stroke. Conditions that may be associated with impaired cognitive functioning but are not specific to older adults such as depression and traumatic brain injury were included if it was clear from the review that it specifically addressed older adults. Reviews covering a population not restricted to older adults were included if the results for older adults were reported separately.

**Types of interventions***.* Reviews were included if they focused on interventions that could be classified as cognitive training, cognitive rehabilitation or cognitive stimulation, according to the definitions provided by Clare and Woods (2004). Reviews focusing largely on combined interventions such as combined cognitive and aerobic training were excluded.

**Types of comparisons.** Reviews were included if the primary trials used an active (e.g. placebo treatment) or passive (e.g. treatment-as-usual, wait-list, no treatment) control condition. Reviews comparing cognition-oriented treatments with other interventions, such as physical exercise, or comparing different types of cognition-oriented treatments were excluded.

**Types of outcomes.** Outcomes included objective cognitive function (global and domain-specific), subjective cognitive function, psychosocial function (e.g. quality of life, mood, depression), functional abilities (activities of daily living), caregiver burden (e.g. burden of care, caregiver stress, caregiver depression), clinical status (e.g. clinical severity and progression or stability rates, rates of admission to residential care, discharge destination) and behavioural and psychological symptoms of dementia. Long-term outcomes were excluded.

### Data Collection and Coding

Data collection was conducted independently by two reviewers (HMG and JS or HH). Disagreements were resolved by consensus and by the involvement of a senior reviewer (AL or ABF) if necessary. For each review, the following data was extracted: review characteristics, details of search strategy and meta-analytic methods, coding of interventions, populations and outcomes, and recording of effect sizes and confidence intervals for each reported outcome. When reviews included a mixture of populations or interventions, we classified them as belonging to the category to which >75% of the included primary trials adhered to. If such a categorization was not possible, they were coded as “mixed population” or “mixed cognition-oriented treatment”. Effect sizes were extracted if they were reported as between-group mean differences and if there was a minimum of two primary trials contributing to the effect size estimate. Reviews that did not provide sufficient statistical information (i.e., between-group effect size estimates and associated confidence intervals) were synthesized narratively.

### Quality Assessment

Quality assessment was conducted independently by two authors (HMG and HH or JS) using A Measurement Tool to Assess Systematic Reviews 2 (AMSTAR: Shea et al., 2017) and disagreements were solved by consensus or by consulting a senior author (ABF or AL). In the case that an author of the present overview had co-authored an eligible review, they were not involved in the quality assessment of that review. In line with the recommendations by Shea et al. (2017), domains in which weaknesses would critically diminish the validity of a review were identified. The following items were considered critical: Item 4 - adequacy of the literature search, Item 9 - adequate assessment of risk of bias, Item 11- appropriateness of meta-analytic methods, Item 13 – consideration of risk of bias when interpreting the results of the review and, Item 15 - assessment of presence and likely impact of publication bias. Based on the fulfilment of critical and non-critical items, the overall confidence in the results of each review was rated as following (a) high – no or one non-critical weakness, (b) moderate – more than one non-critical weakness, (c) low – one critical flaw with or without non-critical weaknesses, or (d) critically low - more than one critical flaw with or without non-critical weaknesses.

In addition, a total score (0–16) was applied for each review. One point was given for each AMSTAR item if the review met the answer “Yes”, 0.5 point for “Partial Yes” and 0 points for “No”. We made some minor adjustments to the AMSTAR ratings as follow. For Item 2 we excluded the item relating to “justification for any deviations from the protocol.” For Item 4, adherence to “justified publication restriction (e.g., language)” was only required for a full “Yes” and for Item 11, adherence to “justified combining the data in a meta-analysis” was only required if any contra-indication for meta-analysis existed.

### Data Analysis

Analyses were performed using Comprehensive Meta-Analysis version 3 (Biostat, NJ). For each reported outcome, the between-group mean difference or standardized mean difference and associated confidence interval (CI) was entered into the Comprehensive Meta-Analysis software. To yield unified effect size estimates, we recalculated all effect sizes into Hedges’ *g* with a 95% CI. The results were investigated by outcome domain (objective cognitive function, subjective cognitive function, psychosocial function, functional abilities, caregiver outcomes, clinical outcomes and behavioural and psychological symptoms of dementia). When reviews provided more than one effect size estimate per outcome domain, the standardized mean difference and variance for each outcome was combined into a single composite. If a review reported an overall composite measure as well as separate results for different subdomains, the composite measure was preferred. The standardized mean differences for each of the main outcome categories were summarized graphically to provide an overview of the available evidence across the different populations, interventions and outcomes. As stated in the registered protocol, we initially intended to pool the data in a second-order meta-analysis if a sufficient number of reviews were available for a certain population and outcome category. However, to avoid the problem of overlapping primary trials across reviews (H. Cooper & Koenka, 2012; McKenzie & Brennan, 2017), we decided against this approach. Instead, for each outcome category, when there was a minimum of three meta-analyses available for a given population or intervention combination, we calculated the mean standardized mean difference and compared this to the standardized mean difference of the “optimal review”, defined as the review that met as many of the following characteristics as possible (1) recent (last 5 years), (2) comprehensive (likely to include most of the relevant literature at the time of publication), (3) rigorous (moderate confidence rating or AMSTAR score > 10). If the mean of the standardized mean differences and the standardized mean difference from the optimal review was reasonably similar, we regarded this as a good approximation of the effect of the intervention on the population and outcome. Using established convention, an effect size of less than 0.2 was considered negligible, between 0.2 and 0.5 small, between 0.5 and 0.8 medium and more than 0.8 large. A sensitivity analysis was also conducted by calculating the mean standardized mean difference while excluding reviews that had a critically low confidence rating. This was done for the main analysis of objective cognitive outcomes, when there was a minimum of three meta-analyses available.

Spearman’s rank correlation was used to investigate the association between AMSTAR score and year of publication. In addition, independent samples *t*-tests were used to compare the mean AMSTAR score of reviews that included only randomized controlled trials (RCTs) versus those that included both RCTs and non-RCTs, and of reviews that had a preregistered protocol versus those that did not. These analyses were performed using IBM SPSS Statistics version 24.

Finally, restricted likelihood, three-level meta-regression models were used to investigate the moderating effect of AMSTAR score, year of publication and number of included studies on overall effect estimate on objective cognitive outcomes. Given many of the included reviews provided more than one effect estimate per outcome for analysis, three-level meta-regression analyses assessed the extent to which the model explains heterogeneity within (${\tau}_{(2)}^2$) and between studies (${\tau}_{(3)}^2$), expressed as ${R}_{(2)}^2$ and ${R}_{(3)}^2$, respectively (Cheung, 2019) . Analyses were conducted using the packages metafor (Viechtbauer, 2010) and metaSEM (Cheung, 2014) on R 3.6.0.

## Results

### Study Selection

**Fig. 1:**
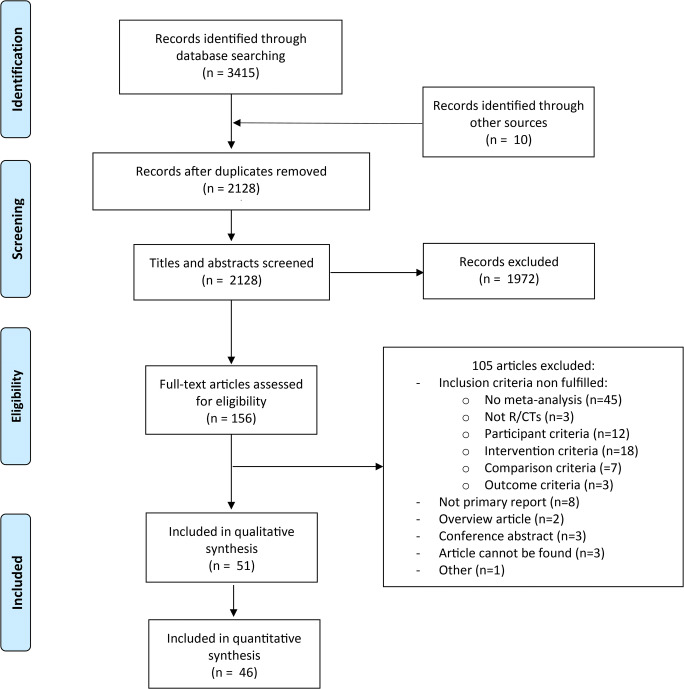
PRISMA flow chart

After duplicate search results were removed, 2128 records were initially screened for eligibility, out of which 1972 were excluded based on titles and abstracts. Of the 156 articles assessed in the full-text screening, 105 were excluded. The most common reason for exclusion was that the review did not include a meta-analysis, followed by not covering the target population or intervention (see Supplementary Material 2 for the full reasons for exclusion). A total of 51 meta-analyses fulfilled inclusion criteria (Fig. 1).

Three reviews (Karbach & Verhaeghen, 2014; Verhaeghen, Marcoen, & Goossens, 1992; Wilson, 2008) did not report effect size estimates as between-group differences, one (Sitzer, Twamley, & Jeste, 2006) did not provide sufficient information for calculation of the CI of the reported effect sizes, and one (Olazaran et al., 2010) reported treatment recommendations. These reviews were therefore only summarized narratively (see Supplementary Material 3). Henceforth, results are presented based on the 46 meta-analyses that provided sufficient information to be included in the quantitative synthesis. A narrative summary of these reviews is provided in Supplementary Material 4.

### Characteristics of Included Studies

The characteristics and methods of the included meta-analyses are presented in Table 1 and Table 2. Out of 46 reviews, 34 reported meta-analytic results for cognitive training, three reported meta-analytic results for cognitive stimulation, three included both cognitive training and cognitive stimulation and six were classified as mixed cognition-oriented treatments. No review reported meta-analytic results for cognitive rehabilitation. A summary of the interventions, populations and outcomes covered by the included meta-analyses is provided in Table 3. Some reviews reported meta-analytic results for more than one population or intervention, which is described in more detail below.

**Table 1:** *Characteristics of Included Meta-Analyses*

Study	Populations included in current analyses	Interventions included in current analyses	Pre-specified control conditions	Outcomes included in current analyses	No. of studies	No. of participants	Age of participants
Alves et al. (2013)	Dementia (AD)	CT	Active	Attention and concentration	4	133	Not reported
				Delayed auditory/verbal memory			
				Delayed verbal recognition			
				Delayed visuospatial memory			
				Immediate auditory/verbal memory			
				Immediate visuospatial memory			
				Global cognition (screening)			
				Verbal fluency phonemic			
				Verbal fluency semantic			
Bahar-Fuchs et al. (2019)	Dementia (mild to moderate, any subtype)	CT	Wait-list, no treatment/standard treatment, active control or alternative treatment (not included in current analyses)	Global cognition (composite)	33	1926	Not reported
				Metacognition (informant-reported)			
				Metacognition (self-reported)			
				General health and QoL			
				Mood			
				ADL			
				Burden of care			
				Disease progression			
				BPSD			
Bhome et al. (2018)	Healthy OA (with subjective cognitive decline)	CT	Active and non-active	Global cognition (composite)	20 (11 CT)	Not reported	Not reported
				Metacognition Psychological well-being			
Chandler et al. (2016)	MCI	Mixed COT	Not reported	Metacognition	30	2093	Not reported
				Mood			
				QoL			
				ADL			
Chiu et al. (2017)	Healthy OA	CT	Any form of control group	Attention	31	6003	65.1–85.1
				Executive function			
				Global cognition (screening)			
				Memory			
				Visuospatial ability			
Cooper et al. (2012)	Dementia	CS	Not reported	QoL	20 (3 CS)	1769 (total)	Not reported
das Nair et al. (2016)	Stroke (with memory deficits)	CT (focusing on memory deficits)	Alternative form of treatment or no memory intervention	Comprehensive memory batteries	13	514	31–68
				Verbal memory			
				Subjective memory			
				Mood			
				Functional ability			
Floyd and Scogin (1997)	Mixed (older adults aged 60 and above, without dementia)	CT (memory training)	Not reported	Subjective memory	27	1150	70.6
				Depression			
Folkerts et al. (2017)	Dementia (living in long-term care facilities)	CS and CT	Usual care, waiting list or active control	Global cognition (screening)	27	1341	69.8–87.8
				QoL/well-being			
Gates, Rutjes, et al. (2019)	Healthy OA	CT (computerized)	Active (unguided computer/screen-based tasks), or inactive (no intervention expected to have an effect on cognition)	Episodic memory	8	1183	67–82
				Executive function			
				Global cognition (composite)			
				Speed of processing Working memory			
Gates, Vernooij, et al. (2019)	MCI	CT (computerized)	Active (unguided computer/screen-based tasks), or inactive (no intervention expected to have an effect on cognition)	Episodic memory Executive function Global cognition (screening) Speed of processing Verbal fluency Working memory Depression Functional performance	8	660	70–82
Gross et al. (2012)	Healthy OA	CT (memory training)	Not reported	Memory	35	3797	60–98
Hill et al. (2017)	MCI and dementia (any ethology)	CT (computerized)	Active or passive	Global cognition (composite)	17 MCI 12 dementia	686 MCI 389 dementia	67–81 MCI 66–81 dementia
				Psychosocial functioning			
				ADL			
				ADL instrumental			
Hindin and Zelinski (2012)	Healthy OA	CT	Not reported	Global cognition (composite)	42 (25 CT)	3781 (2765 CT)	69.9 CT
Hoefler (2016)	Mixed (MCI or AD)	CT (computerized)	Not reported	Attention/processing speed	17	494	64–79.9
				Global cognition (screening)			
				Verbal memory			
				Visual memory Working memory and learning			
				Mental health			
				ADL			
				Assessment of dementia			
Hudes et al. (2019)	Healthy OA	CT (memory strategy training)	Not reported	Memory self-efficacy	18	2895	50–99
				Memory strategy use			
				Memory-related affect			
				Perceived memory ability			
				Psychological well-being			
				QoL			
Huntley et al. (2015)	Dementia	CT, CS and mixed COT	Active, non-active or another treatment	Global cognition (screening)	33	Not reported	66.3–85.7
Karr et al. (2014)	Healthy OA and dementia	CT	Waitlist or placebo	Executive function	46 (23 CT)	4124 (2246 CT)	66–86.4 CT
Kelly et al. (2014)	Healthy OA	CT	Not reported	Attention	31	4555	54–99
				Composite measures of cognitive function			
				Delayed recall Immediate recall			
				Processing speed			
				Recognition			
				Working memory			
				Face name recall			
				Paired associates			
				Subjective memory			
Kim et al. (2017)	Dementia	CS	No treatment, usual care, standard treatment	Global cognition (screening)	14	731	71.8–85.3
				Mood			
				QoL			
				ADL			
				BPSD			
Kurz et al. (2011)	Dementia and mixed (MCI and dementia)	CS and CT	Active, passive, medication	Global cognition (screening)	33	1945	Not reported
Lampit et al. (2014)	Healthy OA	CT (computerized)	Active or passive	Global cognition (composite)	51	4885	60–82
Lawrence et al. (2017)	Parkinson’s disease	CT	Not reported	Attention/working memory	14 (11 CT)	480 (406 CT)	59.70–69.65 CT
				Executive function			
				Global cognition (screening)			
				Memory			
				Visuospatial function			
Lee et al. (2019)	Dementia (caregivers)	Mixed COT	Not reported	Caregiver depressive symptoms	31 (5 COT)	4039 (933 COT)	59.0–69.9 COT
I. H. Leung et al. (2015)	Parkinson’s disease	CT	Not reported	Global cognition (composite)	7	272	59.8–69.1
				Depression			
				QoL			
				ADL			
P. Leung et al. (2017)	Dementia (caregivers)	Mixed COT (cognition-based interventions with caregiver involvement)	No treatment, usual care or treatment as usual, with no caregiver involvement	Carer anxiety	8	803 dyads (dementia patients and carers)	70–78.2 patients 56.8–73.8 carers
				Carer burden/stress			
				Carer depressive symptoms			
				Carer QoL			
				Carer/person with dementia relationship			
Loetscher and Lincoln (2013)	Stroke (with attentional deficits)	CT (focusing on attentional deficits)	Alternative treatment (computerised activities with low attentional demands or social activities) or no attentional intervention	Alertness	6	223	49.5–70.2 treatment 49.6–67.7 control
				Divided attention			
				Selective attention			
				Sustained attention			
				Subjective attention			
				Mood			
				QoL			
				Functional abilities			
Martin et al. (2011)	Healthy older adults and MCI	CT (memory training)	Active or no contact	Delayed recall	33 healthy OA 3 MCI	2229 (2116 healthy OA, 113 MCI)	69.90
				Face-name delayed recall			
				Face-name immediate recall			
				Immediate recall			
				Paired associates Short-term memory			
				Visuospatial memory			
Melby-Lervag and Hulme (2016)	Healthy OA	CT (working memory training)	Not reported	Non-verbal reasoning	17	Not reported	Not reported
Metternich et al. (2010)	Healthy OA (with subjective memory complaints or desire to improve memory performance)	CT (memory training)	Not reported	Objective memory	14	920	53–82
				Subjective memory			
				Depressive symptoms			
Mewborn et al. (2017)	Healthy OA and MCI	CT	Active or passive	Global cognition (composite)	97(48 healthy OA,12 MCI)	8783 (total)	63.75–85.13
Papp et al. (2009)	Healthy OA	CT	Not reported	All outcomes	10	4009	Not reported
Pinquart and Sörensen (2001)	Mixed (no specification of cognitive status)	CT	Untreated control group	Clinican-rated depression	122 (9 CT)	Not reported	55–87
				Psychological well-being			
				Self-rated depression			
Rogers et al. (2018)	Stroke	Mixed COT	Treatment as usual, placebo or waitlist control	Global cognition (composite)	22	1098	48–78
Shao et al. (2015)	Healthy OA	CT (computerised)	Not reported	Executive function	12	2008	66–82
				Memory performance			
				Processing speed			
Sherman et al. (2017)	MCI	Mixed COT	Active or passive	Global cognition (composite)	26	876 (training groups)	66–77
Smart et al. (2017)	Healthy OA (with subjective cognitive decline)	CT	Not reported	Global cognition (composite)	9	676	64.9–77.41
Song et al. (2016)	Dementia (AD or vascular dementia)	CT	Not reported	Global cognition (composite)	13	474	72.00–83.47
Tetlow and Edwards (2017)	Healthy OA	CT (commercially available)	Not reported	Attention	21	5201	Not reported
				Executive function			
				Memory			
				Processing speed			
				Reasoning			
				Verbal fluency			
				Visuospatial memory			
				Everyday function			
Toril et al. (2014)	Healthy OA	CT (videogames)	No control group required (only controlled trials included in current analysis)	Global cognition (composite)	20	913	Not reported
C. Wang et al. (2014)	MCI	CT	Not reported	Delayed memory	18 (11 CT)	1125 (330 CT)	68–86 (68–78 CT)
				Executive function			
				Global cognition (screening)			
				Immediate memory			
				Working memory			
*P.* Wang et al. (2016)	Healthy OA	CT (action videogames)	Not reported	Global cognition (composite)	19 (8 healthy OA)	636 (255 healthy OA)	65–74.8 healthy OA
Weicker et al. (2016)	Healthy OA	CT (working memory training)	Not reported	Attention and processing speed	103 (23 healthy OA)	6113 (978 healthy OA)	Not reported
				Cognitive control and executive function			
				Long-term memory			
				Reasoning and intelligence			
				Working memory			
Virk et al. (2015)	Stroke (with attentional deficits)	CT (focusing on attentional deficits)	Not reported	Alternating attention	12 (6 stroke)	584 (237 stroke)	50.5–68.9 stroke
				Divided attention			
				Inhibition			
				Selective attention			
				Sustained attention			
Woods et al. (2012)	Dementia	CS	No treatment, standard treatment or placebo	Global cognition (screening)	15	718	69.8–85.7
				Communication/social interaction			
				Mood (self-reported)			
				Mood (staff-reported)			
				Well-being and QoL			
				ADL			
				Caregiver depression Carer stress/burden			
				Caregiver anxiety			
				Behaviour problem			
Yang et al. (2018)	Mixed (cognitive decline, MCI, dementia)	Mixed COT (memory-focused interventions)	Not reported	Delayed recall	27	2177	75.80
				Global cognition (screening)			
				Immediate recall			
				Learning and memory function			
				Recognition			
				Subjective memory performance			
				Depression			

*Note.* Reviews may include interventions, populations and outcomes that are beyond the scope of this overview. In such cases, only the information of relevance for the current analyses is described. AD = Alzheimer’s dementia; CT = cognitive training; QoL = quality of life; ADL = activities of daily living; BPSD = behavioural and psychological symptoms of dementia; OA = older adults; MCI = mild cognitive impairment; COT = cognition-oriented treatment, CS = cognitive stimulation

**Table 2:** Methods of Included Meta-Analyses

Study	Protocol	Includes only RCTs	Search period covered	Databases searched	Type and method of effect size	Moderator analyses conducted	Method of grading the quality of the evidence
Alves et al. (2013)	No	Yes	From inception to March 2012	PubMed, PsychINFO, The Cochrane Library, EMBASE, metaRegister of Clinical Trials (ISRCTN Register, NIH ClinicalTrials.gov Register—subset of randomized trial records), OVID all EBM Reviews (Cochrane DSR, ACP Journal Club, DARE, CCTR, HTA, NHSEED).	MD and SMD post-treatment difference	No	Cochrane RoB tool
Bahar-Fuchs et al. (2019)	Yes	Yes	Until July 2018	ALOIS	SMD pre-post difference	Yes	Cochrane RoB tool, GRADE
Bhome et al. (2018)	Yes	Yes	Until August 2017	PubMed, Web of Science, Cochrane Systematic Reviews Database, PsycINFO and CIANHL	Hedges’ g post-treatment difference	Yes	Critical Appraisal Skills Programme
Chandler et al. (2016)	No	No	Until October 2015	MEDLINE, PsycINFO	Cohen’s d pre-post difference	Yes	Not reported
Chiu et al. (2017)	No	Yes	Until December 2016	Cochrane, PubMed, EMBASE, MEDLINE, PsycINFO, and CINAHL.	Hedges’ g	Yes	Cochrane RoB tool
C. Cooper et al. (2012)	No	Yes	Unil January 2011	PubMed, Web of Science, and Cochrane systematic reviews	SMD post-treatment difference	No	Checklist from the Critical Appraisal Skills Program
das Nair et al. (2016)	Yes	Yes	Until May 2016	Cochrane Stroke Group Trials Register, Cochrane Central Register of Controlled Trials, MEDLINE (Ovid), EMBASE (Ovid), CINAHL, AMED, PsycINFO, ClinicalTrials.gov, WHO International Clinical Trials Registry Portal, NIHR Clinical Research Network Database, UK CRN Study Portfolio, LILACS, CAB Abstracts, REHABDATA, Stroke Trials Registry, ISRCTN Registry.	SMD	No	Cochrane RoB tool
Floyd and Scogin (1997)	No	No	1970–1994	PsycLIT, Dissertation Abstracts International	Cohen’s d, post-treatment difference	No	“Scale developed by Suydam (1968)”
Folkerts et al. (2017)	No	No (meta-analysis restricted to RCTs)	Until December 2015	PubMed, which is backed by the MEDLINE database, and CENTRAL (Cochrane Central Register of Controlled Trials)	SMD pre-post difference	Yes	Cochrane RoB tool
Gates, Rutjes, et al. (2019)	Yes	Yes	Until March 2018	ALOIS	SMD pre-post difference	No	Cochrane RoB tool, GRADE
Gates, Vernooij, et al. (2019)	Yes	Yes	Until May 2018	ALOIS	SMD pre-post difference	No	Cochrane RoB tool, GRADE
Gross et al. (2012)	No	No	Until January 2010	PsychInfo, PsychLit, PubMed.	SMD pre-post difference	Yes	Not reported
Hill et al. (2017)	Yes	Yes	From inception to July 2016	Medline, Embase, PsychINFO, CINAHL, and CENTRAL.	Hedges’ g pre-post difference	No	Cochrane RoB tool; PEDro-P scale
Hindin and Zelinski (2012)	No	No	Until January 2011	PSYCINFO, Social Gerontology, and MEDLINE.	Cohen’s d pre-post difference	Yes	A 5-point scale adapted from items used in Papp et al. (2009)
Hoefler (2016)	No	No	1980–2015	Supersearch, Google Scholar.	Cohen’s d post-treatment difference	No	‘Assessment of methodological quality (Lipsey & Wilson, 2009)’ - 10 item scale
Hudes et al. (2019)	Yes	Yes	Until October 2018	PsycINFO, MedLine, Cochrane Central Register of Controlled Trials, Cochrane Database of Systematic Reviews.	Cohen’s d	Yes	Effective Public Health Practice Project tool
Huntley et al. (2015)	No	Yes	Until June 2013	Web of Knowledge, Cochrane Collaborative Central Register of Controlled Trials, and PubMed/Medline	Hedges’ g pre-post difference	Yes	Cochrane RoB tool
Karr et al. (2014)	No	No	Until June 2013	PsychInfo, MedLine, CINAHL, PsycArticles and Cochrane Central Register of Controlled Trials	Cohen’s, pre-post difference	Yes	PEDro scale
Kelly et al. (2014)	Yes	Yes	From 2002 to 2012	PubMed, Medline, the Cochrane Library, and ClinicalTrials.gov, Google Scholar.	SMD pre-post difference	No	Cochrane RoB tool
Kim et al. (2017)	No	Yes	1982 to April 2015	PubMed, MEDLINE (1966 to April 2015), Embase (1980 to April 2015), PsychINFO, and Cochrane Reviews Library	Cohen’s d pre-post difference	No	Cochrane RoB tool
Kurz et al. (2011)	No	Yes	Until December 2010	Medline, Science Citations Index Expanded	Hedges’ g, post-treatment difference	No	Not reported
Lampit et al. (2014)	Yes	Yes	From inception to July 2014	Medline, Embase, and PsychINFO	Hedges’ g pre-post difference	Yes	Cochrane RoB tool; PEDro scale
Lawrence et al. (2017)	No	No	From inception to May 2016	MEDLINE, PubMed, Wiley Online Library and gray literature (e.g., OpenGrey, NTIS).	Hedges’ g pre-post difference	No	Cochrane RoB tool
Lee et al. (2019)	No	Yes	From 2007 to 2017	MEDLINE (Ovid), CINAHL, PsychInfo.	Cohen’s d	No	Cochrane RoB tool
I. H. Leung et al. (2015)	Yes	Yes	From inception to November 2014	Medline (Ovid), Embase, PsychInfo, CINAHL, and CENTRAL	Hedges’ g pre-post difference	No	Cochrane RoB tool, An adapted version of the PEDro-P scale
P. Leung et al. (2017)	No	Yes	Until December 2015	MEDLINE, Embase,Pubmed, PsycINFO, Alois, Cumulative Index of Nursing and Allied Health Literature, Cochrane Library	Hedges’ g pre-post difference	No	Cochrane RoB tool
Loetscher and Lincoln (2013)	Yes	Yes	Until October 2012	CENTRAL, MEDLINE, Embase, PsychInfo, CINAHL, PsycBITE, REHABDATA, ClinicalTrials.gov, Stroke Trials Registry, Current Controlled Trials	MD and SMD, pre-post difference	No	Cochrane RoB tool
Martin et al. (2011)	Yes	Yes	January 1970to September 2007	CENTRAL, MEDLINE, Embase, PsychInfo, CINAHL, SIGLE, LILACS	MD and SMD, pre-post difference	No	Methodological quality of randomisation assessed as described in Cochrane Handbook
Melby-Lervag and Hulme (2016)	No	No	Not reported	PsychInfo, PsycArticles, Medline and Google Scholar and ERIC	Hedges’ g pre-post difference	No	Not reported
Metternich et al. (2010)	No	Yes	Not reported	MedLine and PsycInfo	Hedges’ g pre-post difference	No	In-house developed
Mewborn et al. (2017)	Yes	Yes	Until May 2016	EBSCOhost onlinedatabases (Academic Search Complete, AgeLine,MEDLINE, PsycARTICLES, Psychology and BehavioralSciences Collection, and PsycINFO).	Hedges’ g pre-post difference	Yes	Cochrane RoB tool
Papp et al. (2009)	No	Yes	From 1992 to December 2007	MEDLINE, Scopus, TheCochrane Collaboration, Dissertation Abstract International, and PsycINFO. Registers - Current Controlled Trials and Clinicaltrials.gov.	SMD post-treatment difference	No	Combination of items from a modified version of the Scale to Assess Scientific Quality of Investigations and Jadad
Pinquart and Sörensen (2001)	No	Not reported	Not reported	MEDLINE, PsycINFO, PSYNDEX	Hedges’ g post-treatment difference	Yes	Quality of research report coded by scale of 1 to 3
Rogers et al. (2018)	Yes	Yes	Until December 2017	AMED, CINAHL, Cochrane Library, EMBASE, MEDLINE, PsycEXTRA, PsycINFO, Science Direct, Scopus.	Hedges’ g post-treatment difference	Yes	PEDro
Shao et al. (2015)	No	Yes	From 2000 to October 2014	Pubmed, EMBASE, Cochrane Library, China Knowledge Resource Integrated Database, Wan Fang Data and Weipu Database for Chinese Technical Periodicals.	SMD	Yes	Cochrane RoB tool
Sherman et al. (2017)	No	Yes	From January 1995 to June 2017	MEDLINE-R, PubMed, Healthstar, Global Health, PSYCH-INFO, Health and Psychological Instruments.	Hedges’ g post-treatment difference	Yes	NIH Quality of Assessment of Controlled Intervention Studies Scale
Smart et al. (2017)	No	No	Until November 2015	CINAHL Complete,Cochrane Central Register of Controlled Trials, MEDLINEwith Full Text, PsycINFO, and PsycARTICLES	Bayesian pre-post difference	No	PEDro
Song et al. (2016)	No	Yes	From January 2001 to April 2015	Cochrane Database, EBSCO(CINAHL), PubMed, ProQuest, and ScienceDirect	Not reported	No	Jadad
Tetlow and Edwards (2017)	Yes	Yes	Until 28 July 2017	Google, PubMed, PsychINFO	Cohen’s d post-treatment difference	Yes	PEDro
Toril et al. (2014)	No	No	From 1986 to 2013	MEDLINE,PsychInfo, and Google Scholar	Cohen’s d pre-post difference	Yes	Not reported
C. Wang et al. (2014)	No	Yes	From January 1990 toJanuary 2014	MEDLINE, EMBASE, the Cochranelibrary, and BIOSIS	MD and SMD	Yes	Jadad and Grade profiler 3.6
P. Wang et al. (2016)	No	No	From January 1986 to July 2015	Web of Sciences, PubMed,EBSCO, PsycNET (PsycINFO, PsycARTICLES)	Cohen’s d pre-post difference	Yes	10-item scale was developed
Weicker et al. (2016)	No	No	Until January 2015	PubMed, OvidSP (PsycINFO/PSYNDEX/Medline)	Hedges’ g post-treatment difference	Yes	Not reported
Virk et al. (2015)	No	Yes	From inception to August2014	MEDLINE, EMBASE, PsycINFO andthe Cochrane Central Register of Controlled Trials(CENTRAL)	Hedges’ g	Yes	Cochrane RoB tool
Woods et al. (2012)	Yes	Yes	Until December 2011	ALOIS which includes - MEDLINE, EMBASE, CINAHL, PsycINFO andLILACS; CENTRAL; trial registers,	MD and SMD pre-post difference	No	Cochrane RoB tool
Yang et al. (2018)	No	Yes	Until May 2017	PubMed, Cochrane Library, Ovid-Medline, CINHAL, PsycINFO, Ageline, Embase, Google Scholar.	Hedges’ g pre-post difference	Yes	Cochrane RoB tool

*Note*. RCT = randomized controlled trial; MD = mean difference; SMD = standardized mean difference; RoB = risk of bias; PEDro = Physiotherapy Evidence Database Rating Scale

**Table 3:**
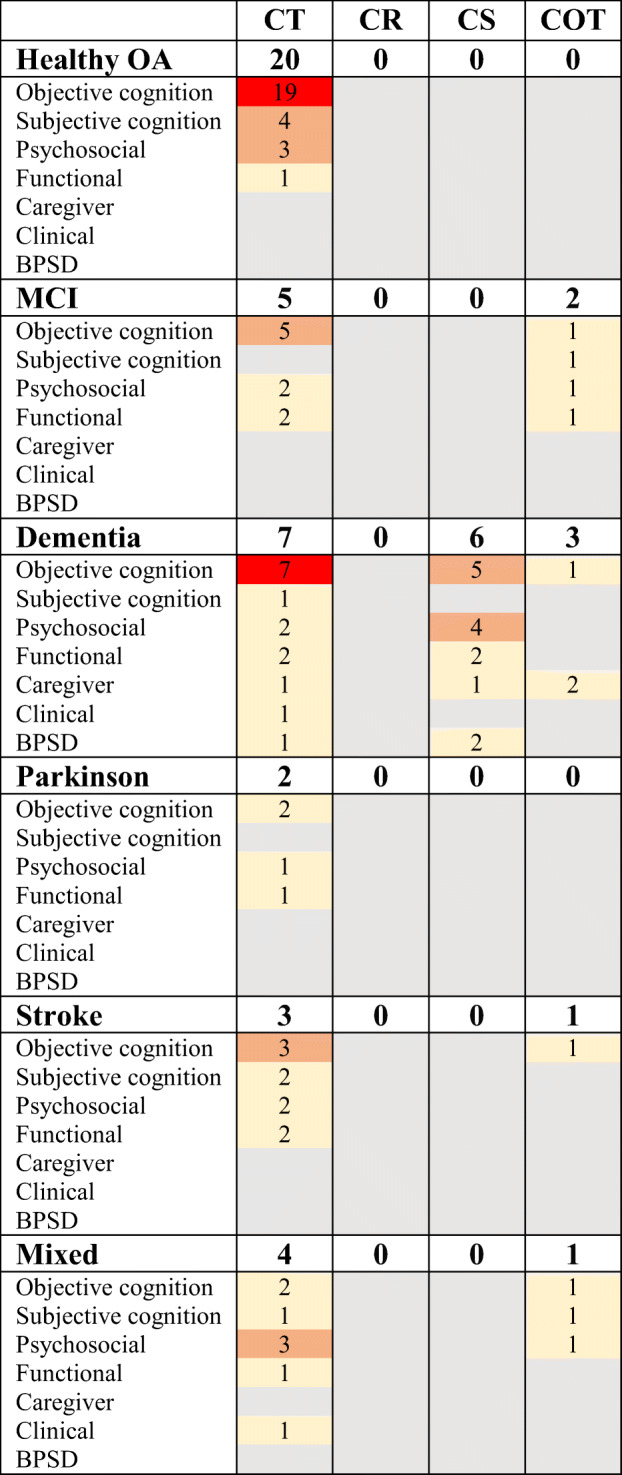
Number of Identified Reviews Reporting Meta-Analytic Results for the Different Types of Interventions, Populations and Outcomes.

*Note*. The number of identified reviews is colour coded: Grey = no reviews; Yellow = 1–2 reviews; Orange = 3–5 reviews; Red= > 5 reviews. CT = cognitive training; CR = cognitive rehabilitation; CS = cognitive stimulation; COT = mixed cognition-oriented treatment; OA = older adults; BPSD = behavioural and psychological symptoms of dementia

**Type of studies.** 32 reviews included only RCTs and 14 reviews included a combination of RCTs and non-RCTs.

**Populations.** Seventeen reviews reported meta-analytic results for healthy older adults (Table 1), three of which focused specifically on individuals with subjective cognitive complaints (Bhome, Berry, Huntley, & Howard, 2018; Metternich, Kosch, Kriston, Harter, & Hull, 2010; Smart et al., 2017). Four reviews focused on individuals with MCI (Chandler, Parks, Marsiske, Rotblatt, & Smith, 2016; Gates et al., 2019; Sherman, Mauser, Nuno, & Sherzai, 2017; Wang et al., 2014). Ten reviews focused on people with dementia (Alves et al., 2013; Bahar-Fuchs et al., 2019; C. Cooper et al., 2012; Folkerts, Roheger, Franklin, Middelstadt, & Kalbe, 2017; Huntley, Gould, Liu, Smith, & Howard, 2015; Kim et al., 2017; Lee et al., 2019; P. Leung, Orgeta, & Orrell, 2017; Song, Lee, & Song, 2016; Woods et al., 2012). One of these reviews included people with dementia living in long-term care facilities (Folkerts et al., 2017). Two reviews focused on PD (Lawrence, Gasson, Bucks, Troeung, & Loftus, 2017; Leung et al., 2015) and four on individuals with stroke (das Nair, Cogger, Worthington, & Lincoln, 2016; Loetscher & Lincoln, 2013; Rogers, Foord, Stolwyk, Wong, & Wilson, 2018; Virk, Williams, Brunsdon, Suh, & Morrow, 2015). Five reviews were classified as a mixed population, three included individuals with MCI and dementia (Hoefler, 2016; Kurz, Leucht, & Lautenschlager, 2011; Yang et al., 2018), one stated that they looked at participants without dementia (Floyd & Scogin, 1997) and one did not specify cognitive status (Pinquart & Sörensen, 2001). Four reviews synthesized pooled effects for more than one population. Two reviews reported meta-analytic results for healthy older adults and MCI (Martin, Clare, Altgassen, Cameron, & Zehnder, 2011; Mewborn, Lindbergh, & Stephen Miller, 2017). One review reported results for healthy older adults and dementia (Karr, Areshenkoff, Rast, & Garcia-Barrera, 2014), and one focused on individuals with MCI and dementia (Hill et al., 2017).

**Interventions.** In all, 34 reviews reported meta-analytic results for cognitive training (Table 1), covering a variety of cognitive training approaches, including computerized training (Gates et al., 2019; Gates, Vernooij, et al., 2019; Hill et al., 2017; Hoefler, 2016; Lampit, Hallock, & Valenzuela, 2014; Shao et al., 2015; Tetlow & Edwards, 2017), memory training (das Nair, Cogger, Worthington, & Lincoln, 2016; Floyd & Scogin, 1997; Gross et al., 2012; Hudes, Rich, Troyer, Yusupov, & Vandermorris, 2019; Martin et al., 2011; Metternich et al., 2010), working memory training (Melby-Lervag & Hulme, 2016; Weicker, Villringer, & Thone-Otto, 2016) and videogames (Toril, Reales, & Ballesteros, 2014; *P.* Wang et al., 2016). Three reviews focused on cognitive stimulation (C. Cooper et al., 2012; Kim et al., 2017; Woods et al., 2012). No review reported meta-analytic results for cognitive rehabilitation. Three reviews reported meta-analytic results for both cognitive training and cognitive stimulation (Folkerts et al., 2017; Huntley et al., 2015; Kurz et al., 2011). Six were classified as mixed cognition-oriented treatments (Chandler et al., 2016; Lee et al., 2019; P. Leung et al., 2017; Rogers et al., 2018; Sherman et al., 2017; Yang et al., 2018), one of which focused specifically on cognition-oriented treatments with caregiver involvement (P. Leung et al., 2017).

### Quality Assessment

**Fig. 2:**
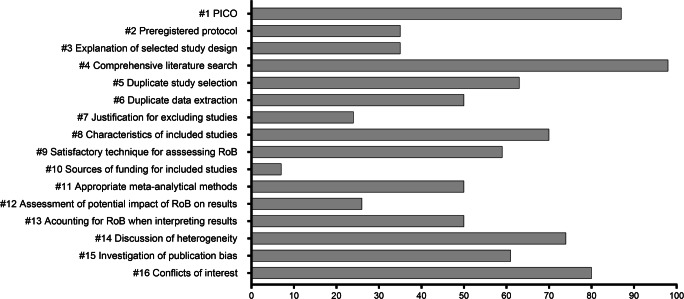
Number of meta-analyses (percent) that adhered to the AMSTAR items AMSTAR = a measurement tool to assess systematic reviews; PICO = population, intervention, comparator group, outcome; RoB = risk of bias.

The results from the AMSTAR ratings for each review are presented in Supplementary Material 5. Mean AMSTAR score was 7.95 (SD = 3.33) out of 16. The confidence ratings were moderate for 9 (20%) of 46 reviews, low for 13 (28%) and critically low for 24 (52%). None of the reviews were classified as high confidence. The number of reviews that adhered to each of the AMSTAR items (i.e., receiving a “Partial Yes” or “Yes”) is shown in Fig. 2. The best adherence was found for conducting a comprehensive literature search (Item 4), using the components of PICO when describing the research question and inclusion criteria (Item 1), reporting conflicts of interest (Item 16) and discussing the impact of heterogeneity on the results (Item 14). The items that most reviews failed to meet were reporting the sources of funding for included studies (Item 10), providing a justification for excluding individual studies (Item 7), and assessing the potential impact of risk of bias on the results (Item 12).

For the critical domains, 45 reviews (98%) conducted a comprehensive literature search (Item 4). Twenty-seven reviews (59%) used a satisfactory technique for assessing the risk of bias in individual studies (Item 9) and 23 reviews (50%) accounted for risk of bias when interpreting the results (Item 13). Twenty-three reviews (50%) adhered to the item of using appropriate methods for statistical combination of the results (Item 11) and 28 reviews (61%) investigated publication bias (Item 15).

There was a significant positive association between AMSTAR score and year of publication (Spearman’s rho = 0.30, *p* = 0.04). However, when restricting the analysis to reviews that were published during the last ten years, thus excluding three older reviews (Floyd & Scogin, 1997; Papp, Walsh, & Snyder, 2009; Pinquart & Sörensen, 2001), there was no significant association (Spearman’s rho = 0.18, *p* = 0.24). Reviews that had a protocol had a higher AMSTAR score [mean 10.31 (SD = 2.90) vs. mean 6.68 (SD = 2.85), t(44) = 4.09, *p* < 0.001], as did reviews that only included RCTs [mean 8.80 (SD = 3.19) vs. mean 6.00 (SD = 2.88), t(44) = 2.81, *p* < 0.01].

### Cognitive Outcomes

**Fig. 3:**
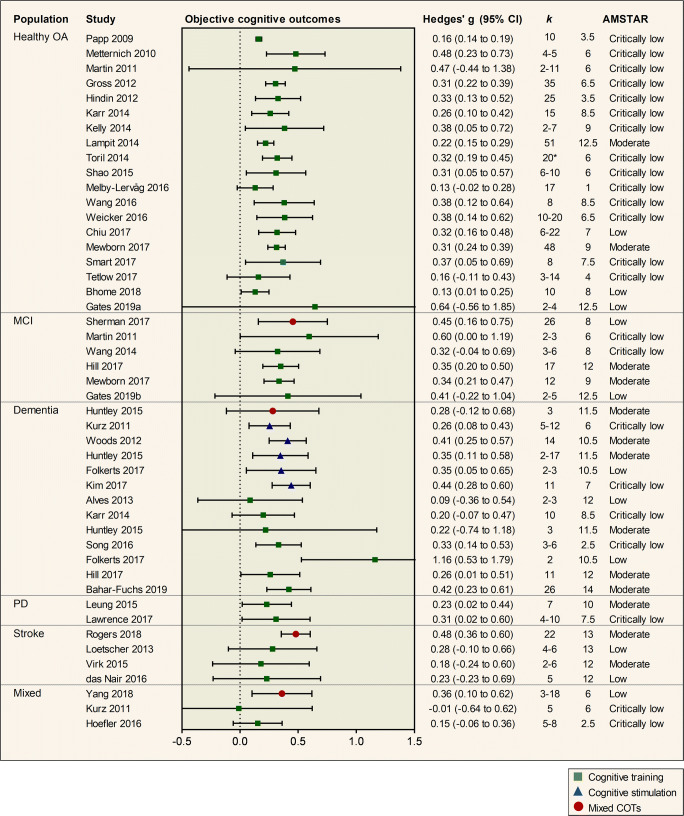
Pooled results of meta-analyses investigating objective cognitive outcomes of cognition-oriented treatments (COT) in older adults. Positive values represent an improvement favouring the intervention group. *k* represents the number of primary trials included in the analysis. If a review reported several effect sizes within each outcome domain, a composite was created and *k* denotes the range of the number of primary trials that contributed to the effect estimate. AMSTAR = a measurement tool to assess systematic reviews (max score 16); OA = older adults; MCI = mild cognitive impairment; PD = Parkinson’s disease.* total number of studies in review.

A total of 39 out of 46 reviews reported at least one pooled effect estimate for objective cognitive outcomes, providing a total of 47 effect estimates (Fig. 3).

Nineteen reviews investigated the effects of cognitive training on objective cognitive outcomes in healthy older adults. The mean effect estimate was 0.32 (0.05 to 0.59). Evidence from the two most comprehensive reviews with moderate confidence ratings showed a small and significant effect in favour of cognitive training on overall cognitive functioning. Lampit et al. (2014) synthesized the results of 51 trials of computerized cognitive training and reported an effect size of Hedges’ *g* = 0.22 (0.15 to 0.29) and Mewborn et al. (2017) pooled the results of 48 trials with an overall effect size of Hedges’ *g* = 0.31 (0.24 to 0.39). In all, 15 of 19 reviews showed a significant intervention effect on objective cognitive outcomes (Fig. 3). In contrast, one review of computerized cognitive training with low confidence AMSTAR rating showed a non-significant effect with large imprecision (Gates, Rutjes, et al., 2019) and three additional reviews with AMSTAR ratings in the critically low range showed no significant effect (Martin et al., 2011; Melby-Lervag & Hulme, 2016; Tetlow & Edwards, 2017).

Five reviews reported meta-analytic results for cognitive training for MCI. The mean effect size estimate was 0.40 (0.03 to 0.78). Evidence from the two most recent and comprehensive reviews with moderate confidence AMSTAR ratings showed a small and significant interventional effect (Hill et al., 2017; Mewborn et al., 2017) with Hedges’ *g* = 0.35 (0.20 to 0.50) and 0.34 (0.21 to 0.47), respectively.

For dementia, five reviews reported meta-analytic results for cognitive stimulation. The mean effect size estimate was 0.36 (0.15 to 0.57). Evidence from the most recent review with a moderate confidence AMSTAR rating by Huntley et al. (2015) showed a small and significant effect of cognitive stimulation on global cognitive screening measures, Hedges’ *g* = 0.35 (0.11 to 0.58). Seven reviews reported meta-analytic results for cognitive training in people with dementia. The mean effect size estimate was 0.38 (−0.04 to 0.80) and the results were heterogeneous (Fig. 3). The most recent and comprehensive review with a moderate confidence rating by Bahar-Fuchs et al. (2019) showed a small and significant effect on cognitive outcomes, Hedges’ *g* = 0.42 (0.23 to 0.61).

Two reviews assessed the effects of cognitive training in PD (Lawrence et al., 2017; Leung et al., 2015), both showed a small and significant interventional effect on cognitive outcomes.

Three reviews reported meta-analytic results for cognitive training for stroke (das Nair et al., 2016; Loetscher & Lincoln, 2013; Virk et al., 2015). The mean effect estimate was 0.23 (−0.19 to 0.65). The most recent review with a moderate confidence AMSTAR rating (Virk et al., 2015) focused specifically on treatment of attentional deficits, showing no significant effect, Hedges’ *g* = 0.18 (−0.24 to 0.60). In contrast, a moderate quality review on mixed cognition-oriented treatments for stroke revealed a small and significant effect on overall cognitive outcomes, Hedges’ *g* = 0.48 (0.36 to 0.60: Rogers et al., 2018). The mean effect estimates were robust to sensitivity analyses, in which reviews with a critically low confidence rating were excluded from the analysis (see Supplementary Material 6).

### Subjective Cognitive Outcomes

**Fig. 4:**
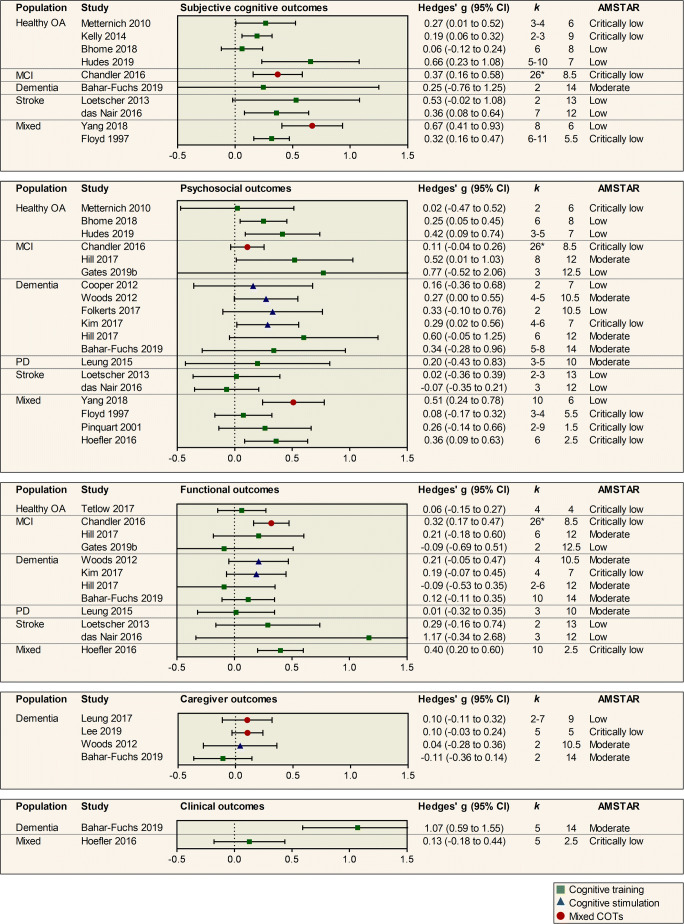
Pooled results of meta-analyses investigating subjective cognitive, psychosocial, functional, caregiver and clinical outcomes of cognition-oriented treatments (COT) in older adults. Positive values represent an improvement favouring the intervention group. *k* represents the number of primary trials included in the analysis. If a review reported several effect sizes within each outcome domain, a composite was created and *k* denotes the range of the number of primary trials that contributed to the effect estimate. AMSTAR = a measurement tool to assess systematic reviews (max score 16); OA = older adults; MCI = mild cognitive impairment; PD = Parkinson’s disease. * total number of studies in review.

Ten out of 46 reviews reported meta-analytic results on subjective cognitive outcomes (Fig. 4). Four reviews evaluated the effects of cognitive training on subjective cognitive complaints in healthy older adults, with confidence ratings in the low (Bhome et al., 2018; Hudes et al., 2019) or critically low range (Kelly et al., 2014; Metternich et al., 2010). The mean effect estimate was 0.29 (0.04 to 0.54). The two most recent reviews with low confidence ratings showed results as follows. Bhome et al. (2018) found no significant effect of cognitive training for individuals with subjective cognitive complaints on metacognition, Hedges’ *g* = 0.06 (−0.12 to 0.24). In contrast, Hudes et al. (2019) investigated the effects of memory-strategy training on a number of meta-memory outcomes, showing a moderate and significant effect, Hedges’ *g* = 0.66 (0.23 to 1.08).

For MCI, the review by Chandler et al. (2016) rated as critically low, demonstrated a small and significant effect of mixed cognition-oriented treatments on metacognitive outcomes. No significant effect of cognitive training on subjective cognition was found for dementia (Bahar-Fuchs et al., 2019). For stroke, one review demonstrated a small and significant improvement favouring cognitive training (das Nair et al., 2016), whereas another review demonstrated a non-significant effect with large imprecision (Loetscher & Lincoln, 2013).

### Psychosocial Outcomes

Eighteen out of 46 reviews assessed psychosocial outcomes, providing 19 effect estimates (Fig. 4). Three reviews assessed the effects of cognitive training on psychosocial outcomes for healthy older adults. The mean effect estimate was 0.23 (−0.11 to 0.57). The two most recent reviews, both rated as a low confidence, showed a small and significant effect on psychosocial outcomes with Hedges’ *g* = 0.25 (0.05 to 0.45) and 0.42 (0.09 to 0.74), respectively (Bhome et al., 2018; Hudes et al., 2019). For MCI, evidence from the most comprehensive, moderate quality review by Hill et al. (2017), showed a moderate significant effect of computerized cognitive training on psychosocial functioning, whereas a non-significant effect with large imprecision was reported in a more recent review rated as low confidence (Gates, Vernooij, et al., 2019). No significant benefits of cognitive training on psychosocial outcomes was found for dementia (Bahar-Fuchs et al., 2019; Hill et al., 2017), PD (I. H. Leung et al., 2015) or stroke (das Nair et al., 2016; Loetscher & Lincoln, 2013).

Four reviews assessed the effects of cognitive stimulation on psychosocial outcomes for dementia. The mean effect size estimate was 0.26 (−0.11 to 0.64). Evidence from one moderate quality review by Woods et al. (2012) showed a borderline significant effect of Hedges’ *g* = 0.27 (−0.004 to 0.55) on the psychosocial composite outcome (Fig. 4). Authors reported significant benefits of cognitive stimulation on quality of life and staff ratings of communication and social interaction, but not for self- or staff-reported mood (Woods et al., 2012).

Four reviews assessed psychosocial outcomes in mixed populations (Floyd & Scogin, 1997; Hoefler, 2016; Pinquart & Sörensen, 2001; Yang et al., 2018), the most recent one was rated as low confidence, showing a moderate effect of mixed cognition-oriented treatments on depressive symptoms for individuals with MCI and dementia (Yang et al., 2018).

### Functional Outcomes

Eleven out of 46 reviews assessed functional outcomes (Fig. 4), providing 12 effect size estimates, one in healthy older adults (Tetlow & Edwards, 2017) three in MCI (mixed cognition-oriented treatments, Chandler et al., 2016; cognitive training, Gates, Vernooij, et al., 2019; Hill et al., 2017), four in dementia (cognitive training, Bahar-Fuchs et al., 2019; Hill et al., 2017; cognitive stimulation, Kim et al., 2017; Woods et al., 2012), one in PD (cognitive training, I. H. Leung et al., 2015), two in stroke (cognitive training, das Nair et al., 2016; Loetscher & Lincoln, 2013) and one in mixed MCI and dementia (cognitive training, Hoefler, 2016). The majority found no significant difference between intervention and control groups on functional abilities. Two reviews with critically low AMSTAR ratings showed contrary results. Chandler et al. (2016) demonstrated a small and significant effect of mixed cognition-oriented treatments on ADL for MCI and Hoefler (2016) showed a small and significant effect of computerized cognitive training on ADL in mixed MCI and dementia.

### Caregiver Outcomes

Four reviews assessed caregiver outcomes, all of which focused on caregivers of people with dementia. One review assessed cognitive stimulation (Woods et al., 2012), one focused on cognitive training (Bahar-Fuchs et al., 2019) and two were classified as mixed cognition-oriented treatments (Lee et al., 2019; Leung et al., 2017). No significant interventional effect was found on the overall caregiver outcomes (Fig. 4). A review with low confidence AMSTAR rating by Leung et al. (2017) reported a small and significant benefit of mixed cognition-oriented treatments with caregiver involvement on carer quality of life and caregiver depressive symptoms, but no significant benefits on caregiver burden, caregiver anxiety or carer to person-with-dementia relationship.

### Clinical Outcomes

Two reviews reported meta-analytic results for clinical outcomes (Fig. 4). A moderate quality review showed a large and significant effect of cognitive training on dementia disease progression with Hedges’ *g* = 1.07 (0.59 to 1.55), however, authors stated that there was uncertainty in the estimate due to large heterogeneity and imprecision (Bahar-Fuchs et al., 2019).

### Behavioural and Psychological Symptoms of Dementia

Three reviews reported meta-analytic results for the effects of cognitive stimulation (Kim et al., 2017; Woods et al., 2012) and cognitive training (Bahar-Fuchs et al., 2019) on behavioural and psychological symptoms in dementia. No significant benefits of the interventions were found (effect sizes ranging from −0.14 to 0.44).

### Meta-Regression

**Fig. 5:**
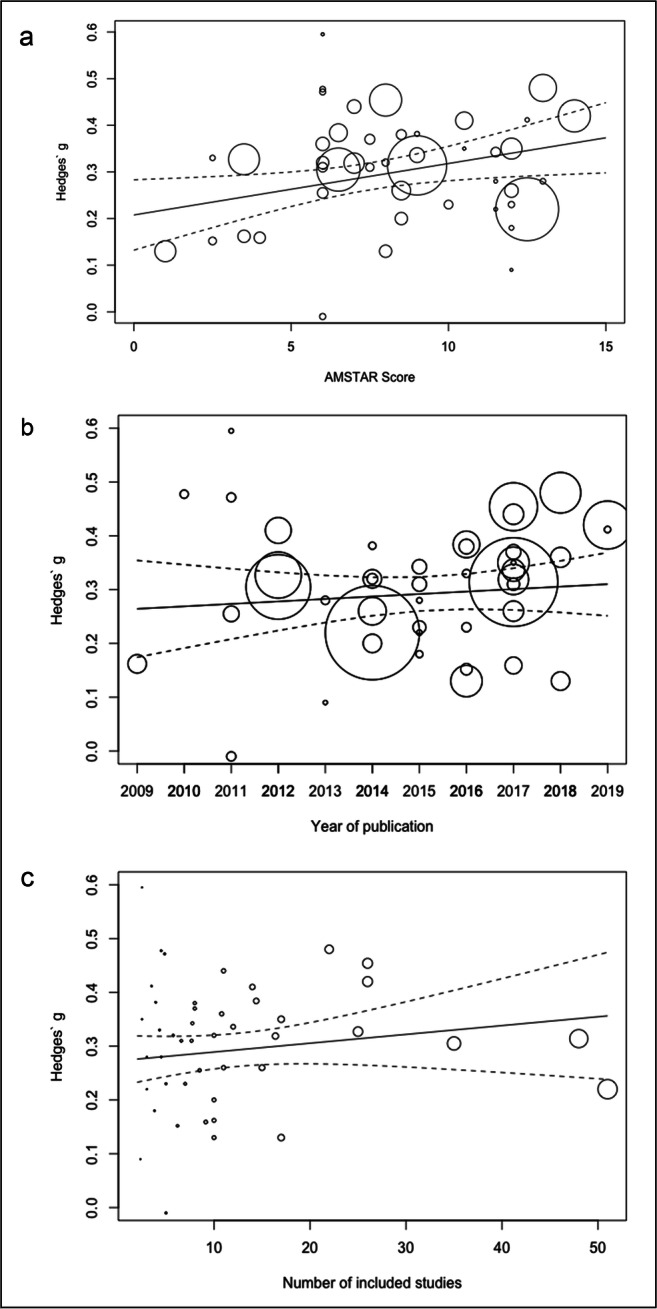
Association between objective cognition effect size and (a) AMSTAR score, (b) year of publication and (c) number of included studies. Circle size refer to the number of included studies. Two extreme effect sizes (Folkerts et al., 2017; Gates, Rutjes, et al., 2019) were omitted from the scatterplots. AMSTAR = a measurement tool to assess systematic reviews.

As expected given substantial overlap of trials across reviews, heterogeneity within reviews (${\tau}_{(2)}^2$= 0.009) was markedly larger than between reviews (${\tau}_{(3)}^2$= 0.0004). Higher AMSTAR score was associated with *larger* effect estimates for objective cognitive outcomes (β = 0.011, 95% CI 0.002 to 0.020, *p* = 0.02, ${R}_{(3)}^2$= 17%, Fig. 5a). There was no evidence for association between effect estimates on cognitive outcomes and year of publication (β = 0.004, 95% CI -0.009 to 0.017, *p* = 0.50, ${R}_{(3)}^2$= 5%) or the number of primary trials included in reviews (β = 0.002, 95% CI -0.001 to 0.004, *p* = 0.26, ${R}_{(2)}^2$= 2%, ${R}_{(3)}^2$= 34%, Fig. 5b and c).

## Discussion

The aim of this overview was to synthesize the available evidence from systematic reviews with meta-analyses investigating the efficacy of the three main types of cognition-oriented treatments for older adults with or without cognitive decline on cognitive and non-cognitive outcomes, while also taking into account the methodological quality of published reviews. We identified 51 reviews that met our inclusion criteria, 46 of which were included in the quantitative synthesis of results.

### Summary of Main Results

There was consistent evidence to support the use of cognitive training for improving cognitive performance in healthy older adults, MCI and PD. For people with dementia, results from published meta-analyses were heterogeneous, however, the most recent and comprehensive review (Bahar-Fuchs et al., 2019) demonstrated moderate-quality evidence for cognitive training improving cognitive performance also in this population. Thus, the potential for cognitive training to improve cognitive outcomes has been demonstrated across the spectrum of cognitive health, from healthy older adults to dementia. We further found moderate-quality evidence to support the use of cognitive stimulation for improving cognitive performance for people with dementia. The mean effect estimates for cognitive training and cognitive stimulation across the different populations suggest modest efficacy. For stroke, we found that meta-analyses of cognitive training interventions targeting a specific cognitive domain, such as memory (das Nair et al., 2016) and attention (Loetscher & Lincoln, 2013; Virk et al., 2015) showed no significant effect, however, results were imprecise and based on a small number of primary trials. In contrast, there was evidence from a moderate-quality review including 22 primary trials covering a variety of cognitive remediation techniques (Rogers et al., 2018), to support the use of cognition-oriented treatments to improve cognitive performance following stroke.

The evidence for cognition-oriented treatments to reduce subjective cognitive complaints was inconsistent, and although several reviews reported benefits on subjective cognitive outcomes, the confidence ratings for these reviews were low or critically low. For psychosocial outcomes, the majority of the reviews found no significant effect. Nevertheless, there was moderate-quality evidence from two reviews to support the efficacy of cognitive training to improve psychosocial functioning in MCI (Hill et al., 2017), and of cognitive stimulation to improve psychosocial functioning in dementia (Woods et al., 2012). There was no convincing evidence supporting the efficacy of cognition-oriented treatments on functional abilities, caregiver outcomes, clinical disease progression or behavioural and psychological symptoms of dementia. Overall, the quality of the evidence for subjective cognition and non-cognitive outcomes was restricted by methodological issues, such as methodological limitations of reviews, a small number of primary trials investigating these outcomes, and imprecise effect estimates.

### Overall Completeness of the Evidence

Of the 46 meta-analyses included in this overview, 20 (43%) reported pooled effect estimates for cognitive training in healthy older adults. Although some of these meta-analyses covered different subtypes of cognitive training approaches and outcome domains, it is still noteworthy that so many meta-analyses addressing the same or closely related questions have been published. Additionally, even though the overall number of primary trials published increases with time, there is no clear indication that the number of trials included in the meta-analyses has increased by year of publication (Fig. 3). Cognitive training has also been thoroughly investigated across older adult populations with cognitive decline, as we identified multiple meta-analyses on cognitive training for MCI, dementia, PD and stroke. For cognitive stimulation, meta-analytic results were only identified for people with dementia. Sherman et al. (2017) included cognitive stimulation in their review of cognitive interventions in MCI but did not identify any eligible trials. Finally, although a protocol for a meta-analysis of cognitive rehabilitation for people with dementia was recently published (Kudlicka et al., 2019), no current reviews were identified that reported meta-analytic results for cognitive rehabilitation, and it was recently concluded that the number of cognitive rehabilitation trials for Alzheimer’s disease (Oltra-Cucarella et al., 2018) and other progressive neurodegenerative disorders (Clare et al., 2018) is still limited. Since cognitive rehabilitation is the cognition-oriented treatment approach that is most directly targeted towards producing functional change (Bahar-Fuchs et al., 2019), future trials exploring the effects of cognitive rehabilitation on functional abilities is an important area of investigation, especially considering the weak evidence for functional improvement following cognitive training and cognitive stimulation.

Cognitive performance was the primary outcome in most meta-analyses, and results for subjective cognition and non-cognitive outcomes were more sparsely reported. Notably, only two reviews reported meta-analytic results for clinical outcomes, such as disease progression. This could be partly due to the fact that we only included post intervention results and that these outcomes are more appropriate to evaluate at follow-up assessments. However, the most recent Cochrane review on cognitive training in dementia identified only two primary trials that reported results for disease progression at follow-up assessments 3 to 12 months post treatment (Bahar-Fuchs et al., 2019). This corroborates the inference that – despite their importance – clinical outcomes are rarely assessed in primary trials. Moreover, although several reviews reported meta-analytic results for subjective cognition, psychosocial and functional outcomes, the pooled effect estimates were generally based on a small number of primary trials and, within these broad categories, a diverse set of outcomes and instruments had been employed. The fact that clinically relevant outcomes are assessed infrequently and with heterogeneous measures has previously been highlighted (Harrison, Noel-Storr, Demeyere, Reynish, & Quinn, 2016; Lees, Fearon, Harrison, Broomfield, & Quinn, 2012) and attempts have been made to increase harmonisation (Webster et al., 2017), which seems imperative in order to improve synthesis efforts in the field of cognition-oriented treatments.

### Methodological Quality of Included Meta-Analyses

The methodological quality of included meta-analyses was highly variable, and we found no clear evidence that methodological standards in reviews have improved in the past 10 years. Strengths included using PICO as an organising framework for formulating the research question, conducting a comprehensive literature search, reporting conflicts of interest and discussing the impact of heterogeneity on the results. However, there were also important limitations. Most reviews did not report the sources of funding for included studies, which could be argued to be particularly important in the field of cognitive training which has been criticized for commercial interests (Stanford Center on Longevity, 2014). Additionally, the majority of reviews did not have a preregistered protocol. Protocol preregistration is important to mitigate bias in the review process (Stewart, Moher, & Shekelle, 2012) and we found that referral to a protocol was associated with higher quality score, over and above the one point given for protocol preregistration. Other concerns were that the majority of reviews did not provide a list of excluded studies and justify their exclusion, nor did they assess the potential impact of risk of bias on the results.

Notably, more than half of the reviews included in this overview received a critically low confidence rating using the AMSTAR tool. This could in part be attributed to the fact that we used fairly wide inclusion criteria and did not restrict our overview to meta-analyses that included only RCTs or to more recent publications. As might be expected, older meta-analyses did not comply to modern standards for meta-analytic methods and reporting and consequently obtained a lower AMSTAR score. Nevertheless, the majority of included meta-analyses were published in the past 10 years and for these, no evidence was found to suggest that review quality improved by year of publication. We further found that higher AMSTAR score was associated with larger cognitive effect sizes. This suggests that further investigation into the relation between the methodological conduct of reviews and their corresponding meta-analytic results might be warranted. Caution has been raised that the rapid increase in published systematic reviews and meta-analyses in the past decades – in some areas even outpacing the number of published RCTs (Niforatos, Weaver, & Johansen, 2019) – has brought about redundant and potentially flawed meta-analyses (Ioannidis, 2016). The results from this overview suggest that a critical appraisal and the implementation of mechanisms to reduce the rate of duplication and redundancy is warranted also in the field of cognition-oriented treatments in older adults.

### Implications for Research

This overview highlights that despite the relatively large evidence-base in the field of cognition-oriented treatments for older adults, there are also areas in which more research is needed. Since we only identified meta-analyses on cognitive stimulation for people with dementia, a future direction for primary trials and subsequent evidence synthesis is to explore the efficacy of cognitive stimulation in other age-related conditions associated with cognitive decline. The effects of cognitive rehabilitation remains largely unexplored, however, a Cochrane review on cognitive rehabilitation in dementia is underway (Kudlicka et al., 2019). Future research on cognitive rehabilitation in other conditions, such as PD and MCI, could also be of value.

It is clear that understanding of the clinical relevance of cognition-oriented treatments is dependent on identifying which outcomes are clinically relevant for patients and caregivers and including these in a more consistent way in future trials. Furthermore, to increase confidence in the available evidence, future meta-analyses should be conducted with rigorous methodological standards and in accordance with available guidelines. Critical areas of improvement include using publicly available and preregistered protocols to reduce the risk of bias in the review process, reporting sources of funding for primary trials, and assessing the impact of risk of bias on the meta-analytic results to confirm their reliability.

Given the wealth of evidence for cognition-oriented treatments improving cognitive outcomes in older adults, evidence synthesis in the field may be most valuable by gradually changing its main focus from questions of mere efficacy to investigating what makes interventions more effective for specific cognition-oriented treatments and target populations. This will require more comprehensive methods than those currently employed in the field, but are nevertheless reasonably developed and have already proved as practice-changing in other clinical fields.

First, the availability of multiple and often overlapping outcome measures within primary trials means that effect sizes are dependent and therefore cannot be simply pooled together in the same analysis. This challenge has so far been handled in various ways, most commonly by selecting a single outcome measure per analysis or by creating composites based on simple means of all available measures of a given outcome. Designs in which data is pooled only when the same outcome measure was used appear to make sense psychometrically, but given limited overlap of outcome measures across studies, this is likely to lead to a large number of small and underpowered analyses, leading to both type I (multiple comparisons) and type II (small sample) errors. Analyses of composite scores of multiple outcome measures might avoid this problem, but may overestimate within-study variance and thus underestimate between-study heterogeneity. Multivariate and multilevel approaches are likely to be more efficient alternatives, as they account for within- and between-study variance and thus allow not only to control for dependency among effect sizes but also to investigate potential sources of variance in each level (for a review, see Cheung, 2019).

Second, investigations of heterogeneity (mixed-effects subgroup analyses and meta-regressions) can be expanded to incorporate network meta-analysis approaches. In addition to attempts to compare different cognition-oriented treatment approaches (e.g., Liang et al., 2018), it may be possible to use component network meta-analysis (Pompoli et al., 2018), which allows for different intervention components to be dismantled and compared in order to identify the optimal combination of intervention ingredients or techniques. In complex interventions such as cognition-oriented treatments, this approach could lead to a better understanding of the most (or least) essential components, which could have important theoretical and clinical implications.

Finally, there is a clear need to understand who is more likely to adhere to and benefit from specific interventions. Individual participant data meta-analyses have the potential to produce fine-grained evidence of the interaction between personal and intervention design factors but are yet to be conducted in the field. As with other more advanced and arguably more clinically informative methods, these types of meta-analyses will require more expansive eligibility criteria aiming to capture clinical and methodological heterogeneity, rather than more restrictive approaches.

### Implications for Practice

The evidence in this overview suggests that cognition-oriented treatments lead to a modest improvement in cognitive performance on standardized measures. Cognition-oriented treatments may be beneficial for subjective cognition and psychosocial functioning, however, the available evidence for benefit was inconsistent and generally of low quality. No convincing evidence suggests that cognition-oriented treatments are associated with benefits on functional abilities, clinical disease progression, caregiver outcomes or behavioural and psychological symptoms of dementia. For many clinically relevant outcomes, the confidence in the available evidence is low, suggesting that the observed effects may change as a result of further, high-quality research. Placing the results from this overview in the context of available treatments for age-associated cognitive decline, the observed benefits of cognition-oriented treatments on cognition are in line with those reported for pharmacological treatments for dementia (Birks & Harvey, 2018), but without, or very rare, adverse effects. Taken together, the available evidence suggests that cognition-oriented treatments are an acceptable approach to maintain cognitive health in old age, while the trade-off between conducting cognition-oriented treatments and participating in other potentially engaging activities also needs to be considered.

### Strengths and Limitations

This overview followed a preregistered protocol and was conducted in accordance with established criteria for systematic reviews. We made efforts to categorize the intervention approach of each included meta-analysis using established definitions (Clare & Woods, 2004) and used a rigorous and detailed instrument for quality assessment (Shea et al., 2017). A broad set of inclusion criteria were applied, in order to identify and synthesize the available evidence on the different types of cognition-oriented treatments on cognitive and non-cognitive outcomes across older adult populations. Thus, this overview provides a comprehensive summary of published meta-analyses in the field, as well as their methodological quality.

Some limitations also need to be addressed. First, the distinction between the different cognition-oriented treatment approaches is not uncomplicated. As has previously been recognized (Bahar-Fuchs et al., 2019; Tardif & Simard, 2011) the different types of cognition-oriented treatments are sometimes used interchangeably in the literature and interventional elements belonging to the different categories can overlap within trials. In our overview, three meta-analyses (das Nair et al., 2016; Loetscher & Lincoln, 2013; Virk et al., 2015) were described as cognitive rehabilitation by review authors, but reclassified as cognitive training in order to be consistent with definitions provided by Clare and Woods (2004). This definition of cognitive rehabilitation emphasizes an individualized approach based on collaborative goal-setting. However, other definitions have also been used, particularly in the field of stroke rehabilitation (Cicerone et al., 2000). Second, since many of the meta-analyses focused on the same intervention or population combination, there is overlap in the primary trials included. This can give a false impression of agreement across meta-analyses and it is important to emphasize that the strength of the evidence is not related to the number of published meta-analyses, but to their comprehensiveness and methodological quality. Third, we used broad intervention and outcome categories and the specific interventions and outcomes included in each category were heterogeneous. Thus, a more detailed investigation of the efficacy of different sub-types of cognition-oriented treatments on specific outcome sub-domains is likely to be informative. However, such an approach was beyond the scope of this overview, as our primary objective was to provide a broad overview of the field. For this reason, we also chose to include meta-analyses that were based on both RCTs and non-RCTs. This could arguably introduce additional sources of bias, since the quality of the evidence from a meta-analysis is always dependent on the methodological quality of included primary trials. However, the inclusion of non-RCTs is accounted for in the quality ratings and consequently also in the overall interpretation of the results. Finally, this overview was restricted to meta-analyses. Thus, this excludes other potentially relevant evidence from systematic reviews without meta-analysis and primary trials.

## Conclusions

This systematic overview showed that cognition-oriented treatments are efficacious in improving cognitive performance in older adults with and without cognitive decline. Whether these effects translate into improvements in clinically meaningful outcomes remains unclear, due to the scarcity of high-quality evidence for outcomes of potential clinical relevance. To establish the clinical usefulness, or lack thereof, of cognition-oriented treatments, an important avenue for future trials is to include relevant non-cognitive outcomes in a more consistent way. We encourage future trials on the efficacy of cognitive rehabilitation across older adult populations, and on cognitive stimulation for non-demented older adults with cognitive decline. For meta-analyses in the field, there is a need for better adherence to methodological standards, and protocol preregistration should be encouraged.

## Electronic supplementary material

ESM 1 (PDF 1574 kb)
