# Supplementary material for: Cognition-Oriented Treatments for Older Adults: a Systematic Overview of Systematic Reviews
Source: Neuropsychol Rev. 2020 Apr 7;30(2):167–93. doi: 10.1007/s11065-020-09434-8 (PMC7305099; doi:10.1007/s11065-020-09434-8)
Supplement: Supplementary file 1 — (PDF 1574 kb) [file 11065_2020_9434_MOESM1_ESM.pdf]

Neuropsychology Review – Supplementary Material

Cognition-oriented treatments for older adults: A systematic overview of systematic reviews

Hanna Malmberg Gavelin [a, b, *], Amit Lampit [b, c], Harry Hallock [c], Julieta Sabatés [b], and Alex Bahar-Fuchs[b]

[a] Department of Psychology, Umeå University, Umeå, Sweden

[b] Academic Unit for Psychiatry of Old Age, University of Melbourne, Melbourne, Australia

[c] Department of Neurology, Charité–Universitätsmedizin Berlin, Berlin, Germany

*Correspondence concerning this article should be addressed to Hanna Malmberg Gavelin, Department of Psychology, Umeå University, SE-901 87 Umeå, Sweden. E-mail: hanna.malmberg-gavelin@umu.se.

Supplementary Material

Supplementary Material 1. Search strategy

Supplementary Material 2. List of excluded studies

Supplementary Material 3. Narrative summary of reviews excluded from quantitative synthesis

Supplementary Material 4. Narrative summary of reviews included in quantitative synthesis

Supplementary Material 5. AMSTAR ratings

Supplementary Material 6. Results from sensitivity analysis

Supplementary Material 1. Search strategy

Medline by Ovid search strategy

1. ((cognit* or neurocognit* or neuropsycholog* or cognition-oriented or cognition-based or memory or memory skills or non-pharmacolog* or nonpharmacolog* or mental) adj2 (interven* or training* or rehabilitat* or remediat* or stimulat* or activit* or enhanc* or exercis* or restructur*)).mp.
2. (Video gam* or videogam* or wii or computer gam* or virtual reality).mp.
3. 1 or 2
4. (aging or ageing or elder* or older or senior* or geriatric or MCI or mild cognitive impairment or cognitive decline or cognitive impairment or memory impairment or dement* or alzheimer* or amnestic or neurocognitive disorder).mp
5. (systematic review or meta-analy* or meta analy*).mp
6. 3 and 4 and 5

Supplementary Material 2. List of excluded studies

**Study**	**Reason for exclusion**
Aguirre et al. (2013)	Not primary report
Anonymous (2012)	Article cannot be found
Bahar-Fuchs et al. (2013)	Not primary report
Basak and Qin (2018)	Not meta-analysis
Biundo et al. (2017)	Article cannot be found
Bleakley et al. (2015)	Not meta-analysis
Bowen et al. (2011)	Does not meet participant criteria
Bowen et al. (2013)	Does not meet intervention criteria
Brodaty and Arasaratnam (2012)	Does not meet intervention criteria
Brunelle-Hamann et al. (2013)	Conference abstract
Burch (2014)	Not meta-analysis
Butler (2017)	Not primary report
Butler et al. (2018)	Not meta-analysis
Cardoso et al. (2017)	Not meta-analysis
Carrion et al. (2013)	Not meta-analysis
Chalfont et al. (2018)	Not meta-analysis
Choi et al. (2017)	Not meta-analysis
Chung et al. (2013)	Does not meet participant criteria
Couture et al. (2017)	Conference abstract
Couture et al. (2018)	Not meta-analysis
Daviglus et al. (2011)	Does not meet intervention criteria
de Oliveira et al. (2015)	Not meta-analysis
Dockx et al. (2016)	Does not meet intervention criteria
Edwards et al. (2018)	Includes long-term follow-ups and trials not meeting participant criteria
Edwards and Green (2018)	Not primary report
Elliott and Parente (2014)	Does not meet participant criteria
Fowler (2011)	Not primary report
Fritz et al. (2015)	Not meta-analysis
Fukushima et al. (2016)	Not meta-analysis
Garcia-Casal et al. (2017)	Does not meet comparison criteria
Garrido-Pedrosa et al. (2017)	Not meta-analysis
Gates et al. (2019)	Not meta-analysis
Ge et al. (2018)	Not meta-analysis
George et al. (2014)	Not meta-analysis
Guaita and Vitali (2004)	Not meta-analysis
Joan Guardia-Olmos et al. (2012)	Does not meet participant criteria
J. Guardia-Olmos et al. (2012)	Does not meet participant criteria
Hoffmann et al. (2010)	Not meta-analysis
Hong et al. (2015)	Not meta-analysis
Hopper et al. (2013)	Not meta-analysis
Howes et al. (2017)	Does not meet intervention criteria
Howren et al. (2014)	Not meta-analysis
Jamieson et al. (2014)	Does not meet participant criteria
Joubert and Chainay (2018)	Not meta-analysis
Kalbe and Folkerts (2016)	Not meta-analysis
Karssemeijer et al. (2017)	Does not meet intervention criteria

Supplementary Material 2. List of excluded studies

Kennedy et al. (2008)	Does not meet participant criteria
Kim et al. (2014)	Not primary report
Kong et al. (2009)	Does not meet intervention criteria
Lampit et al. (2015)	Not meta-analysis
Langa and Levine (2014)	Not meta-analysis
Langenbahn et al. (2013)	Does not meet participant criteria
Law et al. (2014)	Not meta-analysis
K. E. Laver et al. (2015)	Does not meet intervention criteria
Kate E. Laver et al. (2018)	Does not meet intervention criteria
Lehert et al. (2015)	Not meta-analysis
Lenze and Bowie (2018)	Not meta-analysis
H. Li et al. (2011)	Includes uncontrolled trials
J. Li et al. (2016)	Does not meet intervention criteria
Liang et al. (2018)	Does not meet comparison criteria
Liang et al. (2019a)	Does not meet comparison criteria
Liang et al. (2019b)	Does not meet comparison criteria
Lin et al. (2013)	Overview
Lipardo et al. (2017a)	Not meta-analysis
Lipardo et al. (2017b)	Not meta-analysis
Livingston et al. (2005)	Not meta-analysis
Livingston et al. (2014a)	Not meta-analysis
Livingston et al. (2014b)	Not meta-analysis
Livingston et al. (2017)	Overview
Luijpen et al. (2003)	Does not meet intervention criteria
Mansor et al. (2019)	Does not meet intervention criteria
Marusic et al. (2018)	Does not meet outcome criteria
McPhee et al. (2019)	Not meta-analysis
Merriman et al. (2019)	Does not meet comparison criteria
Miklos et al. (2015)	Does not meet participant criteria
Milman (2015)	Not meta-analysis
Ogawa et al. (2016)	Not meta-analysis
Oltra-Cucarella et al. (2016)	Does not meet comparison criteria
Oltra-Cucarella et al. (2018)	Does not meet comparison criteria
Orrell et al. (2012)	Not meta-analysis
Oren et al. (2014)	Includes uncontrolled trials
O'Shea et al. (2019)	Not meta-analysis
Paiva et al. (2015)	Does not meet participant criteria
Paiva et al. (2016)	Does not meet participant criteria
Park and Ingles (2001)	Does not meet participant criteria
Petersen et al. (2018)	Not meta-analysis
Racine et al. (2017)	Conference abstract
Ramprasad et al. (2019)	Does not meet intervention criteria
Rohling et al. (2009)	Includes uncontrolled trials
Seppi et al. (2019)	Not meta-analysis
Simon et al. (2013)	Not primary report
Smallfield and Heckenlaible (2017)	Not meta-analysis
Stanmore et al. (2017)	Does not meet intervention criteria

Supplementary Material 2. List of excluded studies

Stewart et al. (2018)	Not meta-analysis
Teixeira et al. (2012)	Not meta-analysis
Trivedi et al. (2018)	Not meta-analysis
Valenzuela and Sachdev (2009)	Includes long-term follow-up
Vaportzis et al. (2019)	Does not meet intervention criteria
Vazquez et al. (2018)	Does not meet intervention criteria
Webb et al. (2018)	Not systematic review
Verhaeghen (1994)	Article cannot be found
Williams et al. (2018)	Does not meet intervention criteria
Xu et al. (2013)	Not meta-analysis
Zehnder et al. (2009)	Not primary report
Zhu et al. (2016)	Does not meet intervention criteria

References

Aguirre, E., Woods, R. T., Spector, A., & Orrell, M. (2013). Cognitive stimulation for dementia: a systematic review of the evidence of effectiveness from randomised controlled trials. *Ageing Res Rev, 12*(1), 253-262, doi:10.1016/j.arr.2012.07.001.

Anonymous (2012). 'Mental exercise' boosts cognitive function in dementia. (4), 39-40.

Bahar-Fuchs, A., Clare, L., & Woods, B. (2013). Cognitive training and cognitive rehabilitation for mild to moderate Alzheimer's disease and vascular dementia. *Cochrane Database Syst Rev*(6), CD003260, doi:10.1002/14651858.CD003260.pub2.

Basak, C., & Qin, S. (2018). Virtual cognitive training in healthy aging and mild cognitive impairment. In *Aging, technology and health* (pp. 215-235). San Diego, CA: Elsevier Academic Press; US.

Biundo, R., Fiorenzato, E., & Antonini, A. (2017). Nonmotor Symptoms and Natural History of Parkinson's Disease: Evidence From Cognitive Dysfunction and Role of Noninvasive Interventions. [Chapter]. *International Review of Neurobiology, 133*, 389-415, doi:http://dx.doi.org/10.1016/bs.irn.2017.05.031.

Bleakley, C. M., Charles, D., Porter-Armstrong, A., McNeill, M. D., McDonough, S. M., & McCormack, B. (2015). Gaming for health: a systematic review of the physical and cognitive effects of interactive computer games in older adults. *J Appl Gerontol, 34*(3), NP166-189, doi:10.1177/0733464812470747.

Bowen, A., Hazelton, C., Pollock, A., & Lincoln, N. B. (2013). Cognitive rehabilitation for spatial neglect following stroke. *Cochrane Database Syst Rev*(7), CD003586, doi:10.1002/14651858.CD003586.pub3.

Bowen, A., Knapp, P., Gillespie, D., Nicolson, D. J., & Vail, A. (2011). Non-pharmacological interventions for perceptual disorders following stroke and other adult-acquired, non-progressive brain injury. *Cochrane Database Syst Rev*(4), CD007039, doi:10.1002/14651858.CD007039.pub2.

Brodaty, H., & Arasaratnam, C. (2012). Meta-analysis of nonpharmacological interventions for neuropsychiatric symptoms of dementia. *Am J Psychiatry, 169*(9), 946-953, doi:10.1176/appi.ajp.2012.11101529.

Brunelle-Hamann, L., Simard, M., & Thivierge, S. (2013). Effects of cognitive training on behavioral and psychological symptoms in alzheimer's disease: A systematic review of the literature. (2), 33.

Burch, D. (2014). What Could Computerized Brain Training Learn from Evidence-Based Medicine? (11), no pagination.

Butler, M. (2017). Behavioral interventions to prevent, delay, or slow Alzheimer's disease, mild cognitive impairment, or age related cognitive decline. [Conference Abstract]. *Alzheimer's and Dementia, 13 (7)*, P1213.

Supplementary Material 2. List of excluded studies

Butler, M., McCreedy, E., Nelson, V. A., Desai, P., Ratner, E., Fink, H. A., et al. (2018). Does Cognitive Training Prevent Cognitive Decline?: A Systematic Review. [Research Support, U.S. Gov't, P.H.S.

Systematic Review

Video-Audio Media]. *Annals of Internal Medicine, 168*(1), 63-68, doi:https://dx.doi.org/10.7326/M17-1531.

Cardoso, N. D. O., Argimon, I. I. d. L., & Pereira, V. T. (2017). Electronic games and elderly cognition-A systematic review. [Literature Review; Systematic Review]. *Psicologia desde el Caribe, 34*(2), 139-160.

Carrion, C., Aymerich, M., Bailles, E., & Lopez-Bermejo, A. (2013). Cognitive psychosocial intervention in dementia: a systematic review. *Dement Geriatr Cogn Disord, 36*(5-6), 363-375, doi:10.1159/000354365.

Chalfont, G., Milligan, C., & Simpson, J. (2018). A mixed methods systematic review of multimodal non-pharmacological interventions to improve cognition for people with dementia. *Dementia*, 1471301218795289, doi:https://dx.doi.org/10.1177/1471301218795289.

Choi, S. D., Guo, L., Kang, D., & Xiong, S. (2017). Exergame technology and interactive interventions for elderly fall prevention: A systematic literature review. *Appl Ergon, 65*, 570-581, doi:10.1016/j.apergo.2016.10.013.

Chung, C. S., Pollock, A., Campbell, T., Durward, B. R., & Hagen, S. (2013). Cognitive rehabilitation for executive dysfunction in adults with stroke or other adult non-progressive acquired brain damage. *Cochrane Database Syst Rev*(4), CD008391, doi:10.1002/14651858.CD008391.pub2.

Couture, M., Giguere-Rancourt, A., & Simard, M. (2017). Impact of cognitive interventions on cognitive symptoms in idiopathic Parkinson's disease. 1835.

Couture, M., Giguere-Rancourt, A., & Simard, M. (2018). The impact of cognitive interventions on cognitive symptoms in idiopathic Parkinson's disease: a systematic review. *Aging Neuropsychology & Cognition*, 1-22, doi:https://dx.doi.org/10.1080/13825585.2018.1513450.

Daviglus, M. L., Plassman, B. L., Pirzada, A., Bell, C. C., Bowen, P. E., Burke, J. R., et al. (2011). Risk factors and preventive interventions for Alzheimer disease: state of the science. *Arch Neurol, 68*(9), 1185-1190, doi:10.1001/archneurol.2011.100.

de Oliveira, A. M., Radanovic, M., de Mello, P. C., Buchain, P. C., Vizzotto, A. D., Celestino, D. L., et al. (2015). Nonpharmacological Interventions to Reduce Behavioral and Psychological Symptoms of Dementia: A Systematic Review. *Biomed Res Int, 2015*, 218980, doi:10.1155/2015/218980.

Dockx, K., Bekkers, E. M., Van den Bergh, V., Ginis, P., Rochester, L., Hausdorff, J. M., et al. (2016). Virtual reality for rehabilitation in Parkinson's disease. *Cochrane Database Syst Rev, 12*, CD010760, doi:10.1002/14651858.CD010760.pub2.

Edwards, J. D., Fausto, B. A., Tetlow, A. M., Corona, R. T., & Valdes, E. G. (2018). Systematic review and meta-analyses of useful field of view cognitive training. [Meta-Analysis

Review

Systematic Review]. *Neuroscience & Biobehavioral Reviews, 84*, 72-91, doi:https://dx.doi.org/10.1016/j.neubiorev.2017.11.004.

Edwards, J. D., & Green, S. (2018). Ufov Cognitive Training Enhances Iadl and Reduces Dementia Risk: How Do We Move the Field of Behavioral Interventions Forward? [Conference Abstract]. *Alzheimer's and Dementia, 14 (7 Supplement)*, P1626, doi:http://dx.doi.org/10.1016/j.jalz.2018.06.2972.

Elliott, M., & Parente, F. (2014). Efficacy of memory rehabilitation therapy: a meta-analysis of TBI and stroke cognitive rehabilitation literature. *Brain Inj, 28*(12), 1610-1616, doi:10.3109/02699052.2014.934921.

Supplementary Material 2. List of excluded studies

Fowler, S. B. (2011). Cognition training interventions for healthy older people and older people with mild cognitive impairment. (4), 178-179.

Fritz, N. E., Cheek, F. M., & Nichols-Larsen, D. S. (2015). Motor-Cognitive Dual-Task Training in Persons With Neurologic Disorders: A Systematic Review. *J Neurol Phys Ther, 39*(3), 142-153, doi:10.1097/NPT.0000000000000090.

Fukushima, R. L. M., Carmo, E. G. d., Pedroso, R. d. V., Micali, P. N., Donadelli, P. S., Fuzaro Junior, G., et al. (2016). Effects of cognitive stimulation on neuropsychiatric symptoms in elderly with Alzheimer's disease: A systematic review. *Dementia & Neuropsychologia, 10*, 178-184.

Garcia-Casal, J. A., Loizeau, A., Csipke, E., Franco-Martin, M., Perea-Bartolome, M. V., & Orrell, M. (2017). Computer-based cognitive interventions for people living with dementia: a systematic literature review and meta-analysis. *Aging Ment Health, 21*(5), 454-467, doi:10.1080/13607863.2015.1132677.

Garrido-Pedrosa, J., Sala, I., & Obradors, N. (2017). Effectiveness of cognition-focused interventions in activities of daily living performance in people with dementia: A systematic review. [Literature Review; Systematic Review]. *The British Journal of Occupational Therapy, 80*(7), 397-408, doi:http://dx.doi.org/10.1177/0308022617698166.

Gates, N. J., Rutjes, W. S. A., Di Nisio, M., Karim, S., Chong, L.-Y., March, E., et al. (2019). Computerised cognitive training for maintaining cognitive function in cognitively healthy people in midlife. [Systematic Review]. *Cochrane Database of Systematic Reviews*(3).

Ge, S., Zhu, Z., Wu, B., & McConnell, E. S. (2018). Technology-based cognitive training and rehabilitation interventions for individuals with mild cognitive impairment: a systematic review. *BMC Geriatrics, 18*(1), 213, doi:https://dx.doi.org/10.1186/s12877-018-0893-1.

George, S., Crotty, M., Gelinas, I., & Devos, H. (2014). Rehabilitation for improving automobile driving after stroke. *Cochrane Database Syst Rev*(2), CD008357, doi:10.1002/14651858.CD008357.pub2.

Guaita, A., & Vitali, S. F. (2004). Cognitive rehabilitation and training in the Alzheimer's disease: Facts and fantasies. [Italian]. (5), 395-400.

Guardia-Olmos, J., Esparcia, A. J., Morales, A. U., & Ferre, E. G. (2012). Neuropsychological rehabilitation and quality of life in patients with cognitive impairments: A meta-analysis study in Spanish-speaking populations. [References]. (1), 35-42.

Guardia-Olmos, J., Jarne Esparcia, A., Urzua Morales, A., & Gudayol Ferre, E. (2012). Neuropsychological rehabilitation and quality of life in patients with cognitive impairments: a meta-analysis study in Spanish-speaking populations. *NeuroRehabilitation, 30*(1), 35-42, doi:10.3233/NRE-2012-0725.

Hoffmann, T., Bennett, S., Koh, C. L., & McKenna, K. T. (2010). Occupational therapy for cognitive impairment in stroke patients. *Cochrane Database Syst Rev*(9), CD006430, doi:10.1002/14651858.CD006430.pub2.

Hong, Y. J., Jang, E. H., Hwang, J., Roh, J. H., & Lee, J. H. (2015). The Efficacy of Cognitive Intervention Programs for Mild Cognitive Impairment: A Systematic Review. *Curr Alzheimer Res, 12*(6), 527-542.

Hopper, T., Bourgeois, M., Pimentel, J., Qualls, C. D., Hickey, E., Frymark, T., et al. (2013). An evidence-based systematic review on cognitive interventions for individuals with dementia. *Am J Speech Lang Pathol, 22*(1), 126-145, doi:10.1044/1058-0360(2012/11-0137).

Howes, S. C., Charles, D. K., Marley, J., Pedlow, K., & McDonough, S. M. (2017). Gaming for Health: Systematic Review and Meta-analysis of the Physical and Cognitive Effects of Active Computer Gaming in Older Adults. [Meta-Analysis

Review

Systematic Review]. *Physical Therapy, 97*(12), 1122-1137, doi:https://dx.doi.org/10.1093/ptj/pzx088.

Supplementary Material 2. List of excluded studies

Howren, M. B., Vander Weg, M. W., & Wolinsky, F. D. (2014). Computerized cognitive training interventions to improve neuropsychological outcomes: evidence and future directions. *J Comp Eff Res, 3*(2), 145-154, doi:10.2217/cer.14.6.

Jamieson, M., Cullen, B., McGee-Lennon, M., Brewster, S., & Evans, J. J. (2014). The efficacy of cognitive prosthetic technology for people with memory impairments: a systematic review and meta-analysis. *Neuropsychol Rehabil, 24*(3-4), 419-444, doi:10.1080/09602011.2013.825632.

Joubert, C., & Chainay, H. (2018). Aging brain: the effect of combined cognitive and physical training on cognition as compared to cognitive and physical training alone - a systematic review. [Review

Systematic Review]. *Clinical Interventions In Aging, 13*, 1267-1301, doi:https://dx.doi.org/10.2147/CIA.S165399.

Kalbe, E., & Folkerts, A. K. (2016). [Cognitive Training in Parkinson's Disease - A New Therapy Option?]. *Fortschr Neurol Psychiatr, 84 Suppl 1*, S24-35, doi:10.1055/s-0042-100724.

Karssemeijer, E., Aaronson, J. A. J., Bossers, W. J. W., Smits, T. T., Olde Rikkert, M., & Kessels, R. (2017). Positive effects of combined cognitive and physical exercise training on cognitive function in older adults with mild cognitive impairment or dementia: A meta-analysis. *Ageing Res Rev, 40*, 75-83, doi:10.1016/j.arr.2017.09.003.

Kennedy, M. R., Coelho, C., Turkstra, L., Ylvisaker, M., Moore Sohlberg, M., Yorkston, K., et al. (2008). Intervention for executive functions after traumatic brain injury: a systematic review, meta-analysis and clinical recommendations. *Neuropsychol Rehabil, 18*(3), 257-299, doi:10.1080/09602010701748644.

Kim, K., Han, J. W., Kim, Y. J., Park, J. H., Lee, S. B., Lee, J. J., et al. (2014). COGNITIVE STIMULATION AS A THERAPEUTIC MODALITY FOR EARLY DEMENTIA AND MILD COGNITIVE IMPAIRMENT: A META-ANALYSIS STUDY. *Alzheimer's & Dementia: The Journal of the Alzheimer's Association, 10*(4), P602, doi:10.1016/j.jalz.2014.05.1017.

Kong, E. H., Evans, L. K., & Guevara, J. P. (2009). Nonpharmacological intervention for agitation in dementia: a systematic review and meta-analysis. *Aging Ment Health, 13*(4), 512-520, doi:10.1080/13607860902774394.

Lampit, A., Valenzuela, M., & Gates, N. J. (2015). Computerized Cognitive Training Is Beneficial for Older Adults. *J Am Geriatr Soc, 63*(12), 2610-2612, doi:10.1111/jgs.13825.

Langa, K. M., & Levine, D. A. (2014). The diagnosis and management of mild cognitive impairment: a clinical review. *JAMA, 312*(23), 2551-2561, doi:10.1001/jama.2014.13806.

Langenbahn, D. M., Ashman, T., Cantor, J., & Trott, C. (2013). An evidence-based review of cognitive rehabilitation in medical conditions affecting cognitive function. *Arch Phys Med Rehabil, 94*(2), 271-286, doi:10.1016/j.apmr.2012.09.011.

Law, L. L., Barnett, F., Yau, M. K., & Gray, M. A. (2014). Effects of combined cognitive and exercise interventions on cognition in older adults with and without cognitive impairment: a systematic review. *Ageing Res Rev, 15*, 61-75, doi:10.1016/j.arr.2014.02.008.

Laver, K. E., George, S., Thomas, S., Deutsch, J. E., & Crotty, M. (2015). Virtual reality for stroke rehabilitation. *Cochrane Database Syst Rev*(2), CD008349, doi:10.1002/14651858.CD008349.pub3.

Laver, K. E., Lange, B., George, S., Deutsch, J. E., Saposnik, G., & Crotty, M. (2018). Virtual reality for stroke rehabilitation. [Systematic Review]. *Cochrane Database of Systematic Reviews*(1).

Lehert, P., Villaseca, P., Hogervorst, E., Maki, P. M., & Henderson, V. W. (2015). Individually modifiable risk factors to ameliorate cognitive aging: a systematic review and meta-analysis. *Climacteric, 18*(5), 678-689, doi:10.3109/13697137.2015.1078106.

Lenze, E. J., & Bowie, C. R. (2018). Cognitive Training for Older Adults: What Works? [Editorial]. *Journal of the American Geriatrics Society, 66*(4), 645-647, doi:http://dx.doi.org/10.1111/jgs.15230.

Supplementary Material 2. List of excluded studies

Li, H., Li, J., Li, N., Li, B., Wang, P., & Zhou, T. (2011). Cognitive intervention for persons with mild cognitive impairment: A meta-analysis. *Ageing Res Rev, 10*(2), 285-296, doi:10.1016/j.arr.2010.11.003.

Li, J., Theng, Y. L., & Foo, S. (2016). Effect of Exergames on Depression: A Systematic Review and Meta-Analysis. *Cyberpsychol Behav Soc Netw, 19*(1), 34-42, doi:10.1089/cyber.2015.0366.

Liang, J. H., Li, J. Y., Jia, R. X., Wang, Y. Q., Wu, R. K., Zhang, H. B., et al. (2019a). Comparison of Cognitive Intervention Strategies for Older Adults With Mild to Moderate Alzheimer's Disease: A Bayesian Meta-analytic Review. *Journal of the American Medical Directors Association, 20*(3), 347-355, doi:https://dx.doi.org/10.1016/j.jamda.2018.09.017.

Liang, J. H., Shen, W. T., Li, J. Y., Qu, X. Y., Li, J., Jia, R. X., et al. (2019b). The optimal treatment for improving cognitive function in elder people with mild cognitive impairment incorporating Bayesian network meta-analysis and systematic review. [Review]. *Ageing Research Reviews, 51*, 85-96, doi:https://dx.doi.org/10.1016/j.arr.2019.01.009.

Liang, J. H., Xu, Y., Lin, L., Jia, R. X., Zhang, H. B., & Hang, L. (2018). Comparison of multiple interventions for older adults with Alzheimer disease or mild cognitive impairment: A PRISMA-compliant network meta-analysis. [Meta-Analysis]. *Medicine, 97*(20), e10744, doi:https://dx.doi.org/10.1097/MD.0000000000010744.

Lin, J. S., O'Connor, E., Rossom, R. C., Perdue, L. A., & Eckstrom, E. (2013). Screening for cognitive impairment in older adults: A systematic review for the U.S. Preventive Services Task Force. *Ann Intern Med, 159*(9), 601-612, doi:10.7326/0003-4819-159-9-201311050-00730.

Lipardo, D. S., Aseron, A. M. C., Kwan, M. M., & Tsang, W. W. (2017a). Effect of Exercise and Cognitive Training on Falls and Fall-Related Factors in Older Adults With Mild Cognitive Impairment: A Systematic Review. *Arch Phys Med Rehabil, 98*(10), 2079-2096, doi:10.1016/j.apmr.2017.04.021.

Lipardo, D. S., Aseron, A. M. C., Kwan, M. M., & Tsang, W. W. (2017b). Effect of Exercise and Cognitive Training on Falls and Fall-Related Factors in Older Adults With Mild Cognitive Impairment: A Systematic Review. [Review

Systematic Review]. *Archives of Physical Medicine & Rehabilitation, 98*(10), 2079-2096, doi:https://dx.doi.org/10.1016/j.apmr.2017.04.021.

Livingston, G., Johnston, K., Katona, C., Paton, J., Lyketsos, C. G., & Old Age Task Force of the World Federation of Biological, P. (2005). Systematic review of psychological approaches to the management of neuropsychiatric symptoms of dementia. *Am J Psychiatry, 162*(11), 1996-2021, doi:10.1176/appi.ajp.162.11.1996.

Livingston, G., Kelly, L., Lewis-Holmes, E., Baio, G., Morris, S., Patel, N., et al. (2014a). Non-pharmacological interventions for agitation in dementia: systematic review of randomised controlled trials. *Br J Psychiatry, 205*(6), 436-442, doi:10.1192/bjp.bp.113.141119.

Livingston, G., Kelly, L., Lewis-Holmes, E., Baio, G., Morris, S., Patel, N., et al. (2014b). A systematic review of the clinical effectiveness and cost-effectiveness of sensory, psychological and behavioural interventions for managing agitation in older adults with dementia. *Health Technol Assess, 18*(39), 1-226, v-vi, doi:10.3310/hta18390.

Livingston, G., Sommerlad, A., Orgeta, V., Costafreda, S. G., Huntley, J., Ames, D., et al. (2017). Dementia prevention, intervention, and care. [Review]. *The Lancet, 390*(10113), 2673-2734, doi:http://dx.doi.org/10.1016/S0140-6736%2817%2931363-6.

Luijpen, M. W., Scherder, E. J., Van Someren, E. J., Swaab, D. F., & Sergeant, J. A. (2003). Non-pharmacological interventions in cognitively impaired and demented patients--a comparison with cholinesterase inhibitors. *Rev Neurosci, 14*(4), 343-368.

Mansor, N. S., Chow, C. M., & Halaki, M. (2019). Cognitive effects of video games in older adults and their moderators: a systematic review with meta-analysis and meta-regression. *Aging & Mental Health*, 1-16, doi:https://dx.doi.org/10.1080/13607863.2019.1574710.

Marusic, U., Verghese, J., & Mahoney, J. R. (2018). Cognitive-Based Interventions to Improve Mobility: A Systematic Review and Meta-analysis. [Review]. *Journal of the American Medical*

Directors Association, 19(6), 484-491.e483, doi:https://dx.doi.org/10.1016/j.jamda.2018.02.002.

McPhee, G. M., Downey, L. A., & Stough, C. (2019). Effects of sustained cognitive activity on white matter microstructure and cognitive outcomes in healthy middle-aged adults: A systematic review. [Review]. *Ageing Research Reviews, 51*, 35-47, doi:https://dx.doi.org/10.1016/j.arr.2019.02.004.

Merriman, N. A., Sexton, E., McCabe, G., Walsh, M. E., Rohde, D., Gorman, A., et al. (2019). Addressing cognitive impairment following stroke: systematic review and meta-analysis of non-randomised controlled studies of psychological interventions. *BMJ Open, 9*(2), e024429, doi:https://dx.doi.org/10.1136/bmjopen-2018-024429.

Miklos, Z., Mychailyszyn, M., & Parente, R. (2015). The efficacy of cognitive rehabilitation therapy: A meta-analytic review of traumatic brain injury and stroke cognitive language rehabilitation literature. *American Journal of Psychiatry and Neuroscience, 3*(2), 15-22.

Milman, L. H. (2015). Cognitive interventions for Primary Progressive Aphasia: Promising initial results highlight the need for more systematic research targeting impairment, function, and quality of life. *Evidence-Based Communication Assessment and Intervention, 9*(4), 127-133, doi:10.1080/17489539.2016.1155844.

O'Shea, D. M., De Wit, L., & Smith, G. E. (2019). Doctor, Should I Use Computer Games to Prevent Dementia? *Clinical Gerontologist, 42*(1), 3-16, doi:http://dx.doi.org/10.1080/07317115.2017.1370057.

Ogawa, E. F., You, T., & Leveille, S. G. (2016). Potential Benefits of Exergaming for Cognition and Dual-Task Function in Older Adults: A Systematic Review. *J Aging Phys Act, 24*(2), 332-336, doi:10.1123/japa.2014-0267.

Oltra-Cucarella, J., Ferrer-Cascales, R., Clare, L., Morris, S. B., Espert, R., Tirapu, J., et al. (2018). Differential effects of cognition-focused interventions for people with Alzheimer's disease: A meta-analysis. [Meta-Analysis]. *Neuropsychology, 32*(6), 664-679, doi:https://dx.doi.org/10.1037/neu0000449.

Oltra-Cucarella, J., Perez-Elvira, R., Espert, R., & Sohn McCormick, A. (2016). Are cognitive interventions effective in Alzheimer's disease? A controlled meta-analysis of the effects of bias. *Neuropsychology, 30*(5), 631-652, doi:10.1037/neu0000283.

Oren, S., Willerton, C., & Small, J. (2014). Effects of spaced retrieval training on semantic memory in Alzheimer's disease: a systematic review. *J Speech Lang Hear Res, 57*(1), 247-270, doi:10.1044/1092-4388(2013/12-0352).

Orrell, M., Woods, B., & Spector, A. (2012). Should we use individual cognitive stimulation therapy to improve cognitive function in people with dementia? *BMJ, 344*, e633, doi:10.1136/bmj.e633.

Paiva, S., Magalhaes, R., Alves, J., & Sampaio, A. (2015). Efficacy of cognitive intervention in stroke: A long road ahead. *Restor Neurol Neurosci, 34*(1), 139-152, doi:10.3233/RNN-150590.

Paiva, S., Magalhaes, R., Alves, J., & Sampaio, A. (2016). Efficacy of cognitive intervention in stroke: A long road ahead. *Restorative Neurology and Neuroscience, 34*(1), 139-152, doi:10.3233/Rnn-150590.

Park, N. W., & Ingles, J. L. (2001). Effectiveness of attention rehabilitation after an acquired brain injury: a meta-analysis. *Neuropsychology, 15*(2), 199-210.

Petersen, R. C., Lopez, O., Armstrong, M. J., Getchius, T. S. D., Ganguli, M., Gloss, D., et al. (2018). Practice guideline update summary: Mild cognitive impairment report of theguideline development, dissemination, and implementation. *Neurology, 90*(3), 126-135, doi:http://dx.doi.org/10.1212/WNL.0000000000004826.

Racine, E., Plourde, M., & Simard, M. (2017). Working memory training in healthy elderly: A meta-analysis. [Conference Abstract]. *Alzheimer's and Dementia, 13 (7)*, P525.

Ramprasad, C., Tamariz, L., Garcia-Barcena, J., Nemeth, Z., & Palacio, A. (2019). The Use of Tablet Technology by Older Adults in Health Care Settings-Is It Effective and Satisfying? A Systematic Review and Meta Analysis. *Clinical Gerontologist, 42*(1), 17-26, doi:https://dx.doi.org/10.1080/07317115.2017.1322162.

Supplementary Material 2. List of excluded studies

Rohling, M. L., Faust, M. E., Beverly, B., & Demakis, G. (2009). Effectiveness of cognitive rehabilitation following acquired brain injury: a meta-analytic re-examination of Cicerone et al.'s (2000, 2005) systematic reviews. *Neuropsychology, 23*(1), 20-39, doi:10.1037/a0013659.

Seppi, K., Ray Chaudhuri, K., Coelho, M., Fox, S. H., Katzenschlager, R., Perez Lloret, S., et al. (2019). Update on treatments for nonmotor symptoms of Parkinson's disease-an evidence-based medicine review. [Review]. *Movement Disorders, 34*(2), 180-198, doi:http://dx.doi.org/10.1002/mds.27602.

Simon, S. S., Yokomizo, J., Balardin, J. B., Miotto, E. C., & Bottino, C. (2013). Effects of cognitive training, as measured with fMRI, in mild cognitive impairment: A brief review. (4 suppl. 1), P298.

Smallfield, S., & Heckenlaible, C. (2017). Effectiveness of Occupational Therapy Interventions to Enhance Occupational Performance for Adults With Alzheimer's Disease and Related Major Neurocognitive Disorders: A Systematic Review. *Am J Occup Ther, 71*(5), 7105180010p7105180011-7105180010p7105180019, doi:10.5014/ajot.2017.024752.

Stanmore, E., Stubbs, B., Vancampfort, D., de Bruin, E. D., & Firth, J. (2017). The effect of active video games on cognitive functioning in clinical and non-clinical populations: A meta-analysis of randomized controlled trials. *Neurosci Biobehav Rev, 78*, 34-43, doi:10.1016/j.neubiorev.2017.04.011.

Stewart, C., Subbarayan, S., Paton, P., Gemmell, E., Abraha, I., Myint, P. K., et al. (2018). Non-pharmacological interventions for the improvement of post-stroke activities of daily living and disability amongst older stroke survivors: A systematic review. [Meta-Analysis

Research Support, Non-U.S. Gov't]. *PLoS ONE [Electronic Resource], 13*(10), e0204774, doi:https://dx.doi.org/10.1371/journal.pone.0204774.

Teixeira, C. V., Gobbi, L. T., Corazza, D. I., Stella, F., Costa, J. L., & Gobbi, S. (2012). Non-pharmacological interventions on cognitive functions in older people with mild cognitive impairment (MCI). *Arch Gerontol Geriatr, 54*(1), 175-180, doi:10.1016/j.archger.2011.02.014.

Trivedi, D. P., Braun, A., Dickinson, A., Gage, H., Hamilton, L., Goodman, C., et al. (2018). Managing behavioural and psychological symptoms in community dwelling older people with dementia: 1. A systematic review of the effectiveness of interventions. *Dementia*, 1471301218762851, doi:https://dx.doi.org/10.1177/1471301218762851.

Valenzuela, M., & Sachdev, P. (2009). Can cognitive exercise prevent the onset of dementia? Systematic review of randomized clinical trials with longitudinal follow-up. *Am J Geriatr Psychiatry, 17*(3), 179-187, doi:10.1097/JGP.0b013e3181953b57.

Vaportzis, E., Niechcial, M. A., & Gow, A. J. (2019). A systematic literature review and meta-analysis of real-world interventions for cognitive ageing in healthy older adults. [Review]. *Ageing Research Reviews, 50*, 110-130, doi:https://dx.doi.org/10.1016/j.arr.2019.01.006.

Vazquez, F. L., Otero, P., Garcia-Casal, J., Blanco, V., Torres, A. J., & Arrojo, M. (2018). Efficacy of video game-based interventions for active aging. A systematic literature review and meta-analysis. [Literature Review; Systematic Review; Meta Analysis]. *PLoS ONE Vol 13(12), 2018, ArtID e0208192, 13*(12).

Webb, S. L., Loh, V., Lampit, A., Bateman, J. E., & Birney, D. P. (2018). Meta-Analysis of the Effects of Computerized Cognitive Training on Executive Functions: a Cross-Disciplinary Taxonomy for Classifying Outcome Cognitive Factors. [Meta-Analysis

Research Support, Non-U.S. Gov't

Review]. *Neuropsychology Review, 28*(2), 232-250, doi:https://dx.doi.org/10.1007/s11065-018-9374-8.

Verhaeghen, P. (1994). Memory strategy training in the elderly: Possibilities and limits. [Dutch]. (4), 5-14.

Williams, F., Moghaddam, N., Ramsden, S., & De Boos, D. (2018). Interventions for reducing levels of burden amongst informal carers of persons with dementia in the community. A systematic

review and meta-analysis of randomised controlled trials. *Aging & Mental Health*, 1-14, doi:https://dx.doi.org/10.1080/13607863.2018.1515886.

Xu, X. D., Ren, H. Y., Prakash, R., Vijayadas, & Kumar, R. (2013). Outcomes of neuropsychological interventions of stroke. *Ann Indian Acad Neurol, 16*(3), 319-328, doi:10.4103/0972-2327.116909.

Zehnder, F., Martin, M., Altgassen, M., & Clare, L. (2009). Memory training effects in old age as markers of plasticity: a meta-analysis. *Restor Neurol Neurosci, 27*(5), 507-520, doi:10.3233/RNN-2009-0491.

Zhu, X., Yin, S., Lang, M., He, R., & Li, J. (2016). The more the better? A meta-analysis on effects of combined cognitive and physical intervention on cognition in healthy older adults. *Ageing Res Rev, 31*, 67-79, doi:10.1016/j.arr.2016.07.003.

Supplementary Material 3. Narrative summary of reviews that were excluded from the quantitative synthesis of results

Study	Description of review	Key findings	Recommendations for research	Recommended intervention characteristics
Karbach and Verhaeghen (2014)	49 studies evaluated the effect of executive control and working memory training in healthy younger and older adults.<br><br>AMSTAR score: 4.5<br>Confidence rating: critically low	Process-based cognitive training of executive function and working memory was found to have a small to medium effect in healthy older adults. Effect sizes in near and far transfer were small to medium. No differences were found between younger and older adults, adaptive or non-adaptive training, or active and passive controls.	More research is required to investigate the durability and generalizability of training effects to everyday life. Individual differences in effectiveness should be studied, as well as the effects of this type of training on brain structure and function.	
Olazaran et al. (2010)	This review on non-pharmacological interventions for Alzheimer's disease included 179 RCTs. Among these, 14 were cognitive training, and 10 were cognitive stimulation.<br><br>AMSTAR score: 4.5<br>Confidence rating: critically low	Cognitive training and cognitive stimulation were found to be beneficial for cognition. Cognitive stimulation was also shown to improve behavioural outcomes and caregiver psychological well-being. However, these conclusions were made on the basis of low-quality RCTs.	Future high quality, larger, RCTs should include neglected populations (e.g. non-native language speakers or medically ill persons), blinded outcome assessors, and investigate response predictors, including the dose of each intervention component.	
Sitzer et al. (2006)	17 studies including a total of almost 600 participants, investigated the effects of cognitive training on people with Alzheimer's disease.<br><br>AMSTAR score: 3<br>Confidence rating: critically low	Cognitive training may produce benefits on different cognitive and functional outcomes in people with Alzheimer's disease, with effect sizes ranging from small to large. The larger effects were found in relation to executive functions, learning and activities of daily living.	Future, larger studies should investigate whether treatment effects can be generalised to everyday behaviours. IADL measures based on performance should be included for this purpose. Research should also focus on the economic impact of cognitive training for this population, and on its effects on caregivers.	Cognitive training should include caregiver involvement and be delivered in combination with pharmacotherapy.

Supplementary Material 3. Narrative summary of reviews that were excluded from the quantitative synthesis of results

Verhaeghen et al. (1992)	This review, that included controlled and non-controlled trials, examined the effects of mnemonic training on memory performance of cognitively unimpaired older adults. 33 studies with 1539 participants were included.<br><br>AMSTAR score: 3.5<br>Confidence rating: critically low	Mnemonic training was shown to be more beneficial than passive and active control conditions. Being younger, receiving pre-training, group format and shorter sessions were found to be associated with larger benefits.	Further research should include relevant outcome assessment at baseline and investigate the positive effects of group format training and of pre-training.	
Wilson (2008)	This review investigated the effects of memory training on meta-memory of healthy older adults. 17 studies were included with a total of 1163 participants.<br><br>AMSTAR score: 4.5<br>Confidence rating: critically low	Memory training had a small but significant effect on subjective memory, which was larger than the effect of placebo/control groups. Expectancy change was found to be more efficient than traditional memory training after removal of outliers.	More studies are required to investigate generalisability and long-term maintenance of treatment effects, as well as the potential moderators of these effects. Multifactorial interventions and interventions using technology should be included in future research, which should be applied to real-world situations.	Clinicians are encouraged to increase their knowledge on memory training, and this type of intervention should be included in residential homes and community and senior centres.

AMSTAR = A MeaSurement Tool to Assess systematic Reviews ; RCT = randomized controlled trial

Supplementary Material 3. Narrative summary of reviews that were excluded from the quantitative synthesis of results

References

Karbach, J., & Verhaeghen, P. (2014). Making working memory work: a meta-analysis of executive-control and working memory training in older adults. *Psychological Science, 25*(11), 2027-2037, doi:10.1177/0956797614548725.

Olazaran, J., Reisberg, B., Clare, L., Cruz, I., Pena-Casanova, J., Del Ser, T., et al. (2010). Nonpharmacological therapies in Alzheimer's disease: a systematic review of efficacy. *Dementia and Geriatric Cognitive Disorders, 30*(2), 161-178, doi:10.1159/000316119.

Sitzer, D. I., Twamley, E. W., & Jeste, D. V. (2006). Cognitive training in Alzheimer's disease: a meta-analysis of the literature. *Acta Psychiatrica Scandinavia, 114*(2), 75-90, doi:10.1111/j.1600-0447.2006.00789.x.

Verhaeghen, P., Marcoen, A., & Goossens, L. (1992). Improving memory performance in the aged through mnemonic training: a meta-analytic study. *Psychology and Aging, 7*(2), 242-251, doi:10.1037/0882-7974.7.2.242

Wilson, K. Y. (2008). *The effectiveness of memory training programs in improving the subjective memory characteristics of healthy older adults with memory complaints: A meta-analysis*. Doctoral dissertation, Marshall University, Retrieved from Marshall Digital Scholar. (Paper 165).

Supplementary Material 4. Narrative summary of reviews that were included in the quantitative synthesis of results

Study	Description of review	Key findings	Recommendations for research	Recommended intervention characteristics
Alves et al. (2013)	Four randomized controlled trials analyzed the effects of cognitive intervention on people with a diagnosis of dementia due to Alzheimer's disease. Evaluated outcomes included cost-effectiveness of these interventions, global cognition and specific cognitive domains, and psychosocial outcomes, among others.	This review found modest effects on global cognition, but not on the specific cognitive domains. Cognitive interventions were shown to have high rates of completion and adherence.	More high-quality studies utilizing more sensitive and standardized measures are needed. Functional neuroimaging should also be used in these studies. They should include active control conditions that are as comparable to the experimental conditions as possible. The cost-effectiveness of these types of interventions should be investigated.	The authors recommend cognitive intervention as a complementary option to antidementia drugs.
Bahar-Fuchs, Martyr, Goh, Sabates, and Clare (2019)	Thirty-three studies investigated the effect of cognitive training on cognitive and non-cognitive outcomes of people with mild-to-moderate dementia, and on non-cognitive outcomes of their caregivers, compared to active and passive control conditions, as well as to alternative forms of treatment.	Cognitive training probably has small to moderate positive effects on global cognition and on verbal category fluency at the end of treatment, compared to an active or passive control condition. There were improvements in many other domains, but certainty in these findings is low due to the quality of the evidence. When compared to other treatments, no strong evidence of any benefits of cognitive training was found, but certainty in these findings is also low.	High quality and prospectively registered trials with published protocols are required to compare cognitive training to other interventions, explore cost-effectiveness and dose-response-related issues, as well as maintenance of waning of treatment effects. When possible, they should include blinding of participants. Clinically relevant outcomes should be assessed using standardized measures.	
Bhome, Berry, Huntley, and Howard (2018)	Twenty studies evaluated cognitive, psychological, lifestyle and pharmacological interventions for older adults with subjective cognitive decline. Eleven of these focused on cognitive training interventions.	Cognitive training was shown to have a small but significant effect on cognitive outcomes and on psychological well-being, but not on global cognitive performance. However, the quality of the evidence was very poor, and therefore the validity of such findings remains uncertain.	Future higher-quality studies investigating interventions for persons with and without dementia, separately, are needed.	

Supplementary Material 4. Narrative summary of reviews that were included in the quantitative synthesis of results

Study	Description of review	Key findings	Recommendations for research	Recommended intervention characteristics
Chandler, Parks, Marsiske, Rotblatt, and Smith (2016)	The effect of different cognition-oriented interventions (including computerized, therapist-based and multimodal) on mood, meta-cognition, activities of daily living and quality of life, were compared with control conditions in 30 studies with 2093 participants with mild cognitive impairment.	According to the available evidence, cognitive interventions may have the potential for positive impact in persons with mild cognitive impairment. This type of interventions in mild cognitive impairment may be beneficial for everyday life, although further research is required on this matter. Effect size was small but statistically significant.	Social factors, everyday functioning, benefits of combining different interventions, and efficacy in relation to specific intervention characteristics should be investigated in randomized controlled trials with larger samples.	The authors recommend combining physical and cognitive interventions.
Chiu et al. (2017)	Thirty-one studies with a total of approximately 6000 cognitively healthy older adults compared the effects of cognitive training on overall cognitive function, memory, attention, executive function, and visual-spatial abilities, to those of active and passive control conditions.	Existing evidence suggests that cognitive-based training is effective for the healthy elderly, as it can enhance overall cognitive function and executive function with moderate effect, and some other cognitive functions with small effect.	Further research with longer follow-ups is required, and social factors that predict participant outcomes need to be investigated.	The authors recommend at least 3 sessions per week for at least 8 weeks and total training session at least 24 sessions.
Cooper et al. (2012)	Twenty studies of non-pharmacological interventions for people with dementia were included in this review. Three of these were cognitive stimulation interventions.	Group cognitive stimulation was shown a small effect on quality of life of people with dementia living in care facilities immediately after the intervention, but this was not true for individual cognitive stimulation, or for this intervention delivered to community-dwellers with dementia.	The long-term effects of group cognitive stimulation therapy for care home residents with dementia should be investigated. Further research should evaluate the efficacy and cost-effectiveness of interventions aimed at increasing the quality of life of people living with dementia, and greater consensus on how to define and rate quality of life is needed.	

Supplementary Material 4. Narrative summary of reviews that were included in the quantitative synthesis of results

Study	Description of review	Key findings	Recommendations for research	Recommended intervention characteristics
das Nair, Cogger, Worthington, and Lincoln (2016)	Thirteen studies including a total of 514 participants, evaluated the effects of memory training and rehabilitation on subjective and objective memory and other non-cognitive outcomes of people who had experienced stroke.	Memory rehabilitation was shown to have small to moderate effects on subjective reports of memory at the end of treatment, but no effect was found for objective memory, mood, quality of life and functional abilities.	Further, higher quality randomized controlled trials of memory rehabilitation for people who have experienced stroke are needed.	
Floyd and Scogin (1997)	Twenty-seven studies with a total of 1150 participants were included in this review, which investigated the effects of memory training on mental health and subjective memory functioning of older adults with and without dementia.	Memory training was shown to have a small effect on subjective memory functioning, and no effect on depression and mental health. However, the effect of mnemonic interventions did not differ significantly from those of active control conditions (expectancy modification or placebo treatments). Combined mnemonic training and expectancy modification programs yielded the greatest effects.	The included studies should be replicated. They should measure subjective and objective outcomes and try to assess the effects of each of the components of the treatment. Follow-up assessments are also required.	Memory training should also include pretraining, and it should be accompanied by expectancy modification. People's attitudes towards age-related memory loss should be addressed by these memory training programs.
Folkerts, Roheger, Franklin, Middelstadt, and Kalbe (2017)	Twenty-seven studies evaluated the effects of various cognition-oriented interventions in 1341 older adults with dementia and compared them to passive or active control conditions.	Cognitive interventions are secure and effective for persons with dementia living in long-term care facilities. Small to moderate effects showed that cognitive benefits can be assigned to this type of interventions, although it is not clear whether effects on BPSD and quality of life can be attributed to them or they reflect changes due to additional attention.	More studies including active control conditions, with longer follow-ups and using standardized outcome measures should aim to investigate cost-effectiveness, clinical significance, everyday functioning, dose-response relationships, and the difference in the effects of the different types of interventions on institutionalized and non-institutionalized people.	

Supplementary Material 4. Narrative summary of reviews that were included in the quantitative synthesis of results

Study	Description of review	Key findings	Recommendations for research	Recommended intervention characteristics
Gates, Rutjes, et al. (2019a)	Eight studies with a total of 1183 participants were included in this review, which aimed to assess the effects of at least 12 weeks of computerized cognitive training on cognition of healthy older adults.	A meta-analysis of the included trials either did not show computerized cognitive training to influence cognition for most of the included cognitive outcomes or did not allow the authors to draw conclusions about its effects, due to the low and very low quality of the evidence.	Trials evaluating the effects of longer periods of training and including quality of life, psychiatric symptoms and daily functioning are needed. Standardized, reliable tools should be included to measure cognitive function at the end of treatment and over time. Differential effects of moderators should be investigated. Studies should include active control conditions. Attention should also be paid to the quality of reporting.	
Gates, Vernooij, et al. (2019b)	Eight studies with a total of 660 participants evaluated the effects of at least 12 weeks of computerized cognitive training on maintaining or improving cognition of people with mild cognitive impairment.	Low-quality evidence suggested that computerized cognitive training probably has no effect on processing speed, verbal fluency, or quality of life. The quality of the evidence was too low to draw conclusions for the rest of the included outcomes.	Higher quality trials should explore the long-term effects of computerized cognitive training in people with mild cognitive impairment. Sensitive and reliable outcomes should be chosen, the quality of reporting should be improved, and blinding of participants should be attempted, potentially by the inclusion of active control conditions	
Gross et al. (2012)	Thirty-five studies with a total of 3797 participants assessed the effects of memory training on cognitive performance of cognitively healthy older adults.	Memory training interventions had a small and statistically significant effect on memory, which was greater than the pooled effect of the control groups. Additionally, training on multiple strategies was shown to be moderately associated with larger improvements in memory.	Further research with longer follow-ups is needed. The effects of memory training on other domains, as well as its combination with other interventions, such as pharmacotherapy or exercise should be investigated.	
Hill et al. (2017)	Twenty-nine studies compared the effects of computerized-cognitive training on to those of active and passive controls in a total of 1075 participants with mild cognitive impairment (17 trials) and dementia (12 trials).	Computerized-cognitive training is efficacious on global cognition, select cognitive domains, and psychosocial functioning in mild cognitive impairment. Effects were small to moderate. Computerized-cognitive training is unlikely to be beneficial for people with dementia, but immersive technologies might be useful.	Further research with longer follow-ups and larger samples should investigate the effects of booster sessions, compare different cognitive interventions, and include more executive tasks. The efficacy on conversion to dementia should be studied.	

Supplementary Material 4. Narrative summary of reviews that were included in the quantitative synthesis of results

Study	Description of review	Key findings	Recommendations for research	Recommended intervention characteristics
Hindin and Zelinski (2012)	Forty-two studies encompassing 3781 participants assessed the effects of extended cognitive practice and aerobic exercise interventions on reaction time, executive function and memory of healthy older adults.	Both extended cognitive practice and aerobic fitness training have a small yet significant effect on untrained cognitive tasks, with no significant difference between the two types of intervention. High quality extended practice studies produced larger effect sizes while for aerobic training, lower quality studies produced larger effect sizes.	Further research should intend to identify approaches that encourage participation in cognitively beneficial activities, to investigate benefits in subpopulations with health problems, or with different educational levels or ability.	
Hoefler (2016)	Seventeen studies including a total of almost 500 participants investigated the effect of computerized cognitive training on global cognition, specific cognitive domains, mental health, dementia progression and activities of daily living in older adults with AD or mild cognitive impairment.	Existing evidence suggests that computer-based cognitive training is a cost-effective form of intervention. A small effect size showed that it can delay cognitive decline or decrease the symptomatology in persons with early stage AD or with mild cognitive impairment, although it may not improve the overall fate of the neurodegenerative disease.	More qualitative and quantitative studies with larger diverse samples are needed to investigate the effects of CBCT on quality of life, everyday functioning and caregiver-related outcomes.	
Hudes, Rich, Troyer, Yusupov, and Vandermorris (2019)	This review aimed to investigate the impact of memory-strategy training interventions on healthy older adults, using self-reported outcome measures including meta-memory and well-being. It included 18 studies with a total of 2895 participants.	Available evidence suggests that this type of interventions is effective at improving several meta-memory domains, quality of life, and psychological well-being. No significant effect was found for daily function.	Future studies with better designs are required to include process-based interventions, to identify specific elements of the intervention that might affect efficacy, and to investigate the effects of this type of interventions on everyday function more thoroughly.	

Supplementary Material 4. Narrative summary of reviews that were included in the quantitative synthesis of results

Study	Description of review	Key findings	Recommendations for research	Recommended intervention characteristics
Huntley, Gould, Liu, Smith, and Howard (2015)	Thirty-three studies investigated the effect on global cognition of a cognitive intervention, compared to an active or passive control or an alternative intervention in older adults with dementia.	Cognitive stimulation may have a small to moderate effect on improving the scores on MMSE in persons with dementia, when compared to passive and active controls. It may also improve scores on ADAS-Cog. However, these benefits are generally not clinically significant. Cognitive training or mixed cognitive training and stimulation do not improve general cognition in persons with dementia.	More randomized studies should focus on the comparison of different cognitive interventions. They should blind participants and assessors, include active control conditions, use standardized outcome measures, and assess overall cognition and quality of life.	
Karr, Areshenkoff, Rast, and Garcia-Barrera (2014)	Twenty-three studies evaluated the effects of cognitive training on executive function in healthy older adults or older adults with mild cognitive impairment or dementia.	The findings of this review suggest that cognitive training has benefits on executive functions related to the activities of daily living of older adults. Cognitive training presented a potential advantage over physical exercise at improving this domain. The effect was small but significant for cognitively healthy participants, but it was not significant for those participants with cognitive impairment	Higher quality trials should explore the combination of cognitive and physical exercise and focus on executive-related outcomes.	The authors recommend combining cognitive and physical strategies, holistic behavioral programs and person-centered approaches involving enjoyable social settings.
Kelly et al. (2014)	Thirty-one studies with a total of 4555 participants analyzed the impact of cognitive training and mental stimulation on cognitive and everyday functioning in healthy older adults.	Compared to active controls, cognitive training improves cognitive function and executive functions. Compared to no intervention, cognitive training improves memory and subjective measures of cognitive performance. Results show that mental stimulation can improve memory performance as much as cognitive training.	Further research on mental stimulation is needed. Studies need to include standardized training protocols and measures and compare different cognitive interventions. Longer follow-ups and the inclusion of executive tasks are required.	The authors recommend at least 10 group sessions of adaptive cognitive training programs.

Supplementary Material 4. Narrative summary of reviews that were included in the quantitative synthesis of results

Study	Description of review	Key findings	Recommendations for research	Recommended intervention characteristics
Kim et al. (2017)	Fourteen studies investigated the effect of cognitive stimulation on cognition, mood, behavioral and functional outcomes of people with dementia, compared to passive control conditions.	Cognitive stimulation can be an effective non-pharmacological treatment for improving cognition and quality of life in people with dementia.	More studies are needed to investigate dose-response relationships.	
Kurz, Leucht, and Lautenschlager (2011)	Thirty-three studies encompassing 1945 participants assessed the clinical significance of cognition-focused intervention for older adults with mild cognitive impairment and dementia.	Cognition-focused interventions were shown to have small effects on trained cognitive abilities, comparable to those of antidementia drugs, although only single trials showed significant effects on the delay of cognitive decline, on everyday activities, or on personal goals achievement.	More research including consistent methods should focus on tailoring these types of interventions to individual needs and resources. It should assess person-centered outcomes and implement appropriate treatment duration.	
Lampit, Hallock, and Valenzuela (2014)	Fifty-one randomized controlled trials investigated the effect of computerized-cognitive training on cognitively healthy older adults, compared to active and passive control conditions.	Computerized-cognitive training has a positive, although small, effect on cognitive performance in healthy older adults, but its efficacy varies across cognitive domains and design choices, and it is ineffective for executive functions and verbal memory. Specifically, unsupervised at-home training and training more than three times a week is ineffective.	Further research with better targeted CCT technology should intend to assess the therapeutic responsiveness of verbal memory and executive outcomes, as well as the effects of combining CCT with other interventions, such as physical exercise or memory strategy training.	The authors recommend less than 3 group sessions per week lasting more than 30 minutes, including direct supervision by a trainer, motivational support and encouragement, problem solving of IT issues and social interaction.
Lawrence, Gasson, Bucks, Troeung, and Loftus (2017)	Fourteen trials investigated the effect of cognitive training and non-invasive brain stimulation on cognition of people with Parkinson's disease.	The existing evidence suggests that standard and tailored cognitive training can improve attention/working memory, executive function and memory in people with dementia.	Better design trials with larger samples of people with varying severity of cognitive impairment should investigate the effects of cognitive training on quality of life, everyday functioning, behavioral and affective symptoms, and language. Further research needs to compare different types of cognitive training interventions, and to assess the effects of combining it with brain stimulation, as well as to investigate efficacy in relation to specific intervention characteristics.	

Supplementary Material 4. Narrative summary of reviews that were included in the quantitative synthesis of results

Study	Description of review	Key findings	Recommendations for research	Recommended intervention characteristics
Lee et al. (2019)	Thirty-one trials investigated the effects of the different non-pharmacological interventions available for depressive symptoms among caregivers of people with dementia. 5 of these studies, which a total of 933 participants, included cognitive rehabilitation interventions.	Cognitive rehabilitation was not shown to have an effect on caregivers' depressive symptoms.		
I. H. Leung et al. (2015)	Seven studies with 272 participants with Parkinson's disease were included to assess the effects of cognitive training on cognitive and behavioral outcomes.	Cognitive training appears to be safe and, although modestly, beneficial in cognition of patients with mild to moderate Parkinson's disease, particularly in working memory, executive functioning and processing speed.	Larger, multicenter randomized controlled trials need to investigate the efficacy of CT in secondary prevention of cognitive decline in this population, and to target more cognitively impaired people with Parkinson's disease.	
P. Leung, Orgeta, and Orrell (2017)	Eight studies encompassing 803 dyads studied the effects on caregiver well-being of their involvement in cognitive interventions for people with dementia.	Caregiver involvement in cognition-based interventions for people with dementia has benefits on their quality of life and depressive symptoms, which can contribute to the cost-effectiveness of this type of intervention, according to this review.	Better quality randomized controlled trials and studies with larger samples are required to study this type of interventions. Control groups should also receive cognition=based interventions, but without caregiver involvement.	
Loetscher and Lincoln (2013)	This review focused on cognitive rehabilitation for attention deficits in stroke survivors. It included 6 studies with a total of 223 participants.	No statistically significant effect was found in this review for cognitive rehabilitation on global attention or functional outcomes. However, the included studies suggest that this intervention has a moderate effect on divided attention in the short term.	Higher quality studies with better reporting are needed to investigate persistent effects and the effects on attention applied to everyday life.	The benefits of attention rehabilitation have to be monitored, as there is currently no recommendation of a specific rehabilitation approach.

Supplementary Material 4. Narrative summary of reviews that were included in the quantitative synthesis of results

Study	Description of review	Key findings	Recommendations for research	Recommended intervention characteristics
Martin, Clare, Altgassen, Cameron, and Zehnder (2011)	Thirty-six studies were included in this review on cognition-based interventions for older adults with and without cognitive impairment. Only 3 of these included people with mild cognitive impairment.	Findings suggest that memory training interventions produce significant effects on immediate and delayed verbal memory of healthy older adults, when compared to a no-treatment control condition.	More standardized protocols are needed to maximize comparability between studies. More trials including people with mild cognitive impairment and investigating different types and intensity of existing interventions are required.	
Melby-Lervag and Hulme (2016)	This paper was written in response to Au et al. (2014) and Karbach and Verhaeghen (2014) and reanalyzed the studies reviewed in these papers, focusing on working memory training in adulthood and old age.	Two recent meta-analyses claimed that working memory training was effective in improving cognitive skills in adulthood and stemming cognitive decline in old age. However, there is currently no evidence to produce such statements.	Future studies of working memory should include treated control groups.	
Metternich, Kosch, Kriston, Harter, and Hull (2010)	14 studies investigating the effect of different non-pharmacological interventions on memory, depressive symptoms and well-being of people with subjective memory complaints were included in this review.	Memory training was not shown to be efficient on subjective memory, depression or well-being, but it produced a small effect on objective memory.	Further studies developing and evaluating interventions for subjective memory complaints should measure depressive symptoms and psychological well-being.	Combining interventions, such as memory training with expectancy modification could make it easier to reach the people.
Mewborn, Lindbergh, and Stephen Miller (2017)	Ninety-seven studies with a total of 8783 participants were included in this review of cognitive interventions for cognition of older adults.	Results suggested that cognitive interventions are equally effective for older adults with mild cognitive impairment and cognitively healthy older adults. These interventions can help maintain cognitive functioning, quality of life and independence for older adults.	Future research should investigate the relationship between intervention duration and characteristics and its effects, both immediately after treatment and in the longer term, as well as other moderator variables, such as participant personality and motivation. Additionally, future studies should include standardized measures of cognitive functioning that have previously shown to be robust to practice effects, include more diverse and more impaired populations, and consider the importance of randomization and allocation concealment.	

Supplementary Material 4. Narrative summary of reviews that were included in the quantitative synthesis of results

Study	Description of review	Key findings	Recommendations for research	Recommended intervention characteristics
Papp, Walsh, and Snyder (2009)	Ten randomized controlled trials with a total of 4009 participants were included in this review of cognitive interventions in healthy older adults.	There is currently no evidence that cognitive interventions programs can delay progression to dementia in healthy older adults. However, the ability to compare results between the studies is limited due to methodological issues.	Future studies with longer follow-ups and active control groups should aim to see improvements in more than one domain. Interventions and outcomes should be selected according to neuroscientific evidence.	
Pinquart and Sörensen (2001)	One hundred twenty-three studies of psychotherapeutic or psychosocial interventions for older adults were included in this review.	According to current findings, psychosocial interventions could be effective in improving subjective well-being in healthy older adults and reducing depression in older adults with mental disorders.	Further studies are needed to validate the results from this review.	Improved gerontological and geriatric training for people who work with older adults is recommended.
Rogers, Foord, Stolwyk, Wong, and Wilson (2018)	Twenty-two studies investigated the effectiveness of cognitive remediation in stroke survivals. Outcomes evaluated included global cognition and specific cognitive domains, quality of life, and disability.	According to the findings of this review, cognitive remediation produces small to moderate effects on various domains analyzed, suggesting that this type of intervention is both effective and efficient in this population.	Future high-quality studies are needed to investigate early intervention approaches and whether the effects are maintained in the longer term. Measures evaluating the effect of cognitive remediation on daily life of stroke survivals are required.	
Shao et al. (2015)	Twelve studies analyzed the effects of computerized cognitive training on memory, processing speed and executive function of healthy older adults.	Available evidence suggests that computerized cognitive programs have small to moderate effects on memory performance and processing speed, but no effect on executive function. The improvements on memory can be retained at the longer-term.	More well-designed randomized controlled trials with longer follow-ups are required.	
Sherman, Mauser, Nuno, and Sherzai (2017)	This review on cognitive intervention for people with mild cognitive impairment included 26 studies and investigated the effects of this type of intervention on cognitive outcomes.	Multicomponent training and multidomain-focused strategies produce moderate effects on cognition of people with mild cognitive impairment.		

Supplementary Material 4. Narrative summary of reviews that were included in the quantitative synthesis of results

Study	Description of review	Key findings	Recommendations for research	Recommended intervention characteristics
Smart et al. (2017)	This review on non-pharmacological interventions for people with subjective cognitive decline, included 9 studies, 8 of which were cognitive interventions.	Available evidence suggests that non-pharmacological interventions may be a viable alternative for people with subjective cognitive decline, as well as more cost-effective than medications and less associated with side effects. This review also found that cognitive interventions can result beneficial for objective cognitive functioning.	Future studies should characterize participants adequately and include an estimate of premorbid function and cognitive reserve. Screening measures of psychological functioning should also be included. It is recommended that studies use either a treatment-as-usual passive control or an active control, that they include follow-ups of at least one year, and that novel interventions are standardized and manualized.	Non-pharmacological interventions could be provided as part of preventative care.
Song, Lee, and Song (2016)	Thirteen studies with a total of 474 participants evaluated the effects of cognitive intervention on dementia patients.	Available evidence suggests that multiple cognitive intervention produces a small effect on dementia patients, computer-based intervention produces medium effects, and memory training produces large effects.	Future studies should compare intervention programs that were not included in this review.	Clinicians should consider the order in which cognitive intervention produces effects in people with dementia when selecting the intervention to be delivered: memory training intervention, computer-based intervention, and multiple cognitive intervention.
Tetlow and Edwards (2017)	Twenty-one studies investigated the effects of commercially available computerized cognitive training on cognition and everyday function of cognitively unimpaired older adults.	This review suggests that computerized cognitive training produces small to moderate effects on attention, processing speed, visuospatial memory, and daily function of healthy older adults.	There is a need of meta-analysis investigating the efficacy of individual programs. More research should aim to study the effectiveness of this intervention on everyday function. Longer follow-ups are warranted.	
Toril, Reales, and Ballesteros (2014)	Twenty studies with a total of 913 participants investigated the effects of video game training on cognition of healthy older adults.	Available evidence suggests that video game training produces small to moderate positive effects on global cognition and various specific cognitive skills. Methodological factors, as well as age, moderated the magnitude of the effect.	Future studies should include both active and passive control groups and further investigate the effect moderators. Additionally, research should focus on transfer to cognitive function, especially executive functions, which can be achieved by incorporating neuroimaging data.	

Supplementary Material 4. Narrative summary of reviews that were included in the quantitative synthesis of results

Study	Description of review	Key findings	Recommendations for research	Recommended intervention characteristics
Virk, Williams, Brunsdon, Suh, and Morrow (2015)	Twelve studies with a total of 584 participants investigated the effects of cognitive interventions on attentional deficits following acquired brain injury. Six of these included a total of 237 stroke survivors.	According to current findings, cognitive rehabilitation produces short-term moderate improvements in divided attention after stroke, but these were not evident at follow-up.	Further studies with longer follow-ups are needed to compare different populations, attention domains and rehabilitation approaches.	The cost and resources that are needed to incorporate cognitive rehabilitation interventions into clinical care should be taken into consideration.
C. Wang et al. (2014)	Eighteen studies were included in this review of non-pharmacological interventions for older adults with mild cognitive impairment. Of these, 11 evaluated the effects of cognition-based interventions.	Available evidence suggests that cognition-based interventions produce positive effects on global cognition, executive function, and delayed memory in people with mild cognitive impairment.	Further randomized controlled trials with larger samples are longer interventions are required to investigate the clinical value of these findings and whether effects are maintained over time.	
P. Wang et al. (2016)	This review on the effects of action video game training in healthy adults included 19 studies, 8 of which included older adults.	Current findings suggest that action video game training may improve both overall and specific cognitive functions in healthy adults. Young adults benefited more from action video game than older.	Self-designed action video game should be included in future studies to achieve better effects. Further research including active controls should investigate the effects of this intervention in clinical populations.	
Weicker, Villringer, and Thone-Otto (2016)	One hundred three studies analyzed the effects of working memory training on working memory and other cognitive and non-cognitive domains in various populations. Twenty-three of these studies included a total of 978 healthy older adults.	Overall, working memory training was shown to have a moderate effect on untrained working memory tasks, and a small effect on everyday life functioning. It also showed small effects in other cognitive domains, such as reasoning/intelligence, attention, and delayed memory, some of which were maintained at the longer term.	More high-quality, randomized studies including active control groups are required to investigate the impact of moderator variables.	

Supplementary Material 4. Narrative summary of reviews that were included in the quantitative synthesis of results

Study	Description of review	Key findings	Recommendations for research	Recommended intervention characteristics
Woods, Aguirre, Spector, and Orrell (2012)	Fifteen studies with a total of 718 participants investigated the effectiveness of cognitive stimulation in improving cognitive function in people with dementia.	Cognitive stimulation has small to moderate effects on cognitive function at the end of treatment, self-reported quality of life and well-being at a three-month follow-up, and on communication and social interaction, as rated by the staff.	The long-term benefits of different modalities of cognitive stimulation programmes in people with varying dementia severity should be further studied, as well as the neural processes of cognitive changes and their relationship with mood, quality of life, daily function and behavior, and the clinical meaningfulness of any benefits. Cost-effectiveness, benefits in relation to social inclusion and individual goals, and implementation of these programmes in real-life settings are also areas that require further investigation.	People with mild to moderate dementia should be able to participate in cognitive stimulation programmes.
Yang et al. (2018)	Twenty-seven studies with a total of 2177 participants assessed the effects on memory, global cognition, and depression of memory-focused interventions for people with cognitive disorders, including cognitive decline, mild cognitive impairment, and dementia.	Current findings suggest that memory-focused interventions have small to moderate effects on learning and memory function, subjective memory performance, immediate and delayed recall, global cognitive function, and depression. Individual memory training of shorter duration and more than 8 treatment sessions were shown to produce the largest effects.	Future research should investigate the benefits of these interventions for groups of people at different stages of cognitive disorders and include multiple outcome measures to assess cognitive and non-cognitive domains. Intervention protocols should take participants' age and educational level into consideration, and more training sessions of shorter duration should be included.	Clinical nursing staff can teach participants internal and external memory strategies to improve their memory abilities.

Supplementary Material 4. Narrative summary of reviews that were included in the quantitative synthesis of results

References

Alves, J., Magalhaes, R., Thomas, R. E., Goncalves, O. F., Petrosyan, A., & Sampaio, A. (2013). Is there evidence for cognitive intervention in Alzheimer disease? A systematic review of efficacy, feasibility, and cost-effectiveness. *Alzheimer Disease & Associated Disorders, 27*(3), 195-203. doi:10.1097/WAD.0b013e31827bda55

Au, J., Sheehan, E., Tsai, N., Duncan, G. J., Buschkuehl, M., & Jaeggi, S. (2014). Improving fluid intelligence with training on working memory: a meta-analysis. *22*(2), 366-377.

Bahar-Fuchs, A., Martyr, A., Goh, A. M., Sabates, J., & Clare, L. (2019). Cognitive training for people with mild to moderate dementia. *Cochrane Database of Systematic Reviews, 3,* CD013069. doi:10.1002/14651858.CD013069.pub2

Bhome, R., Berry, A. J., Huntley, J. D., & Howard, R. J. (2018). Interventions for subjective cognitive decline: systematic review and meta-analysis. *BMJ Open, 8*(7), e021610. doi:10.1136/bmjopen-2018-021610

Chandler, M. J., Parks, A. C., Marsiske, M., Rotblatt, L. J., & Smith, G. E. (2016). Everyday impact of cognitive interventions in mild cognitive impairment: a systematic review and meta-analysis. *Neuropsychology Review, 26*(3), 225-251. doi:10.1007/s11065-016-9330-4

Chiu, H. L., Chu, H., Tsai, J. C., Liu, D., Chen, Y. R., Yang, H. L., & Chou, K. R. (2017). The effect of cognitive-based training for the healthy older people: A meta-analysis of randomized controlled trials. *PLoS One, 12*(5), e0176742. doi:10.1371/journal.pone.0176742

Cooper, C., Mukadam, N., Katona, C., Lyketsos, C. G., Ames, D., Rabins, P., . . . Livingston, G. (2012). Systematic review of the effectiveness of non-pharmacological interventions to improve quality of life of people with dementia. *International Psychogeriatrics, 24*(6), 856-870. doi:10.1017/S1041610211002614

das Nair, R., Cogger, H., Worthington, E., & Lincoln, N. B. (2016). Cognitive rehabilitation for memory deficits after stroke. *Cochrane Database of Systematic Reviews, 9,* CD002293. doi:10.1002/14651858.CD002293.pub3

Floyd, M., & Scogin, F. (1997). Effects of memory training on the subjective memory functioning and mental health of older adults: a meta-analysis. *Psychology and Aging, 12*(1), 150-161.

Folkerts, A. K., Roheger, M., Franklin, J., Middelstadt, J., & Kalbe, E. (2017). Cognitive interventions in patients with dementia living in long-term care facilities: Systematic review and meta-analysis. *Archives of Gerontology and Geriatrics, 73,* 204-221. doi:10.1016/j.archger.2017.07.017

Gates, N. J., Rutjes, A. W., Di Nisio, M., Karim, S., Chong, L. Y., March, E., . . . Vernooij, R. W. (2019). Computerised cognitive training for maintaining cognitive function in cognitively healthy people in late life. *Cochrane Database of Systematic Reviews, 3,* CD012277. doi:10.1002/14651858.CD012277.pub2

Gates, N. J., Vernooij, R. W., Di Nisio, M., Karim, S., March, E., Martinez, G., & Rutjes, A. W. (2019). Computerised cognitive training for preventing dementia in people with mild cognitive impairment. *Cochrane Database of Systematic Reviews, 3,* CD012279. doi:10.1002/14651858.CD012279.pub2

Gross, A. L., Parisi, J. M., Spira, A. P., Kueider, A. M., Ko, J. Y., Saczynski, J. S., . . . Rebok, G. W. (2012). Memory training interventions for older adults: a meta-analysis. *Aging & Mental Health, 16*(6), 722-734. doi:10.1080/13607863.2012.667783

Hill, N. T., Mowszowski, L., Naismith, S. L., Chadwick, V. L., Valenzuela, M., & Lampit, A. (2017). Computerized cognitive training in older adults with mild cognitive impairment or dementia: a systematic review and meta-analysis. *American Journal of Psychiatry, 174*(4), 329-340. doi:10.1176/appi.ajp.2016.16030360

Hindin, S. B., & Zelinski, E. M. (2012). Extended practice and aerobic exercise interventions benefit untrained cognitive outcomes in older adults: a meta-analysis. *Journal of the American Geriatrics Society, 60*(1), 136-141. doi:10.1111/j.1532-5415.2011.03761.x

Hoefler, C. E. (2016). *Computer-based cognitive training and Alzheimer's disease: A meta-analysis.* (Doctoral dissertation), Saint Mary's University of Minnesota, Retrieved from ProQuest. (10164041).

Supplementary Material 4. Narrative summary of reviews that were included in the quantitative synthesis of results

Hudes, R., Rich, J. B., Troyer, A. K., Yusupov, I., & Vandermorris, S. (2019). The impact of memory-strategy training interventions on participant-reported outcomes in healthy older adults: A systematic review and meta-analysis. *Psychology and Aging, 34*(4), 587-597. doi:10.1037/pag0000340

Huntley, J. D., Gould, R. L., Liu, K., Smith, M., & Howard, R. J. (2015). Do cognitive interventions improve general cognition in dementia? A meta-analysis and meta-regression. *BMJ Open, 5*(4), e005247. doi:10.1136/bmjopen-2014-005247

Karbach, J., & Verhaeghen, P. (2014). Making working memory work: a meta-analysis of executive-control and working memory training in older adults. *Psychological Science, 25*(11), 2027-2037. doi:10.1177/0956797614548725

Karr, J. E., Areshenkoff, C. N., Rast, P., & Garcia-Barrera, M. A. (2014). An empirical comparison of the therapeutic benefits of physical exercise and cognitive training on the executive functions of older adults: a meta-analysis of controlled trials. *Neuropsychology, 28*(6), 829-845. doi:10.1037/neu0000101

Kelly, M. E., Loughrey, D., Lawlor, B. A., Robertson, I. H., Walsh, C., & Brennan, S. (2014). The impact of cognitive training and mental stimulation on cognitive and everyday functioning of healthy older adults: a systematic review and meta-analysis. *Ageing Research Reviews, 15*, 28-43. doi:10.1016/j.arr.2014.02.004

Kim, K., Han, J. W., So, Y., Seo, J., Kim, Y. J., Park, J. H., . . . Kim, K. W. (2017). Cognitive stimulation as a therapeutic modality for dementia: a meta-analysis. *Psychiatry Investigation, 14*(5), 626-639. doi:10.4306/pi.2017.14.5.626

Kurz, A. F., Leucht, S., & Lautenschlager, N. T. (2011). The clinical significance of cognition-focused interventions for cognitively impaired older adults: a systematic review of randomized controlled trials. *International Psychogeriatrics, 23*(9), 1364-1375. doi:10.1017/S1041610211001001

Lampit, A., Hallock, H., & Valenzuela, M. (2014). Computerized cognitive training in cognitively healthy older adults: a systematic review and meta-analysis of effect modifiers. *PLoS Medicine, 11*(11), e1001756. doi:10.1371/journal.pmed.1001756

Lawrence, B. J., Gasson, N., Bucks, R. S., Troeung, L., & Loftus, A. M. (2017). Cognitive training and noninvasive brain stimulation for cognition in Parkinson's disease: a meta-analysis. *Neurorehabiliation and Neural Repair, 31*(7), 597-608. doi:10.1177/1545968317712468

Lee, M., Ryoo, J. H., Chung, M., Anderson, J. G., Rose, K., & Williams, I. C. (2019). Effective interventions for depressive symptoms among caregivers of people with dementia: A systematic review and meta-analysis. *Dementia*, Epub Jan 12. doi:10.1177/1471301218822640

Leung, I. H., Walton, C. C., Hallock, H., Lewis, S. J., Valenzuela, M., & Lampit, A. (2015). Cognitive training in Parkinson disease: A systematic review and meta-analysis. *Neurology, 85*(21), 1843-1851. doi:10.1212/WNL.0000000000002145

Leung, P., Orgeta, V., & Orrell, M. (2017). The effects on carer well-being of carer involvement in cognition-based interventions for people with dementia: a systematic review and meta-analysis. *International Journal of Geriatric Psychiatry, 32*(4), 372-385. doi:10.1002/gps.4654

Loetscher, T., & Lincoln, N. B. (2013). Cognitive rehabilitation for attention deficits following stroke. *Cochrane Database of Systematic Reviews*(5), CD002842. doi:10.1002/14651858.CD002842.pub2

Martin, M., Clare, L., Altgassen, A. M., Cameron, M. H., & Zehnder, F. (2011). Cognition-based interventions for healthy older people and people with mild cognitive impairment. *Cochrane Database of Systematic Reviews*(1), CD006220. doi:10.1002/14651858.CD006220.pub2

Melby-Lervag, M., & Hulme, C. (2016). There is no convincing evidence that working memory training is effective: A reply to Au et al. (2014) and Karbach and Verhaeghen (2014). *Psychonomic Bulletin & Review, 23*(1), 324-330. doi:10.3758/s13423-015-0862-z

Metternich, B., Kosch, D., Kriston, L., Harter, M., & Hull, M. (2010). The effects of nonpharmacological interventions on subjective memory complaints: a systematic review and meta-analysis. *Psychotherapy and Psychosomatics, 79*(1), 6-19. doi:10.1159/000254901

Supplementary Material 4. Narrative summary of reviews that were included in the quantitative synthesis of results

Mewborn, C. M., Lindbergh, C. A., & Stephen Miller, L. (2017). Cognitive interventions for cognitively healthy, mildly impaired, and mixed samples of older adults: a systematic review and meta-analysis of randomized-controlled trials. *Neuropsychology Review, 27*(4), 403-439. doi:10.1007/s11065-017-9350-8

Papp, K. V., Walsh, S. J., & Snyder, P. J. (2009). Immediate and delayed effects of cognitive interventions in healthy elderly: a review of current literature and future directions. *Alzheimer's and Dementia, 5*(1), 50-60. doi:10.1016/j.jalz.2008.10.008

Pinquart, M., & Sörensen, S. (2001). How effective are psychotherapeutic and other psychosocial interventions with older adults? A meta-analysis. *Journal of Mental Health and Aging, 7*(2), 207-243.

Rogers, J. M., Foord, R., Stolwyk, R. J., Wong, D., & Wilson, P. H. (2018). General and domain-specific effectiveness of cognitive remediation after stroke: systematic literature review and meta-analysis. *Neuropsychology Review, 28*(3), 285-309. doi:10.1007/s11065-018-9378-4

Shao, Y. K., Mang, J., Li, P. L., Wang, J., Deng, T., & Xu, Z. X. (2015). Computer-based cognitive programs for improvement of memory, processing speed and executive function during age-related cognitive decline: a meta-analysis. *PLoS One, 10*(6), e0130831. doi:10.1371/journal.pone.0130831

Sherman, D. S., Mauser, J., Nuno, M., & Sherzai, D. (2017). The efficacy of cognitive intervention in mild cognitive impairment (MCI): a meta-analysis of outcomes on neuropsychological measures. *Neuropsychology Review, 27*(4), 440-484. doi:10.1007/s11065-017-9363-3

Smart, C. M., Karr, J. E., Areshenkoff, C. N., Rabin, L. A., Hudon, C., Gates, N., . . . Wesselman, L. (2017). Non-pharmacologic interventions for older adults with subjective cognitive decline: systematic review, meta-analysis, and preliminary recommendations. *Neuropsychology Review, 27*, 245-257. doi:10.1007/s11065-017-9342-8

Song, Y. W., Lee, J. S., & Song, A. Y. (2016). Meta-analysis about cognitive intervention effect applied to dementia patients. *NeuroRehabilitation, 39*(2), 319-327. doi:10.3233/NRE-161363

Tetlow, A. M., & Edwards, J. D. (2017). Systematic literature review and meta-analysis of commercially available computerized cognitive training among older adults. *Journal of Cognitive Enhancement, 1*(4), 559-575. doi:10.1007/s41465-017-0051-2

Toril, P., Reales, J. M., & Ballesteros, S. (2014). Video game training enhances cognition of older adults: a meta-analytic study. *Psychology and Aging, 29*(3), 706-716. doi:10.1037/a0037507

Virk, S., Williams, T., Brunsdon, R., Suh, F., & Morrow, A. (2015). Cognitive remediation of attention deficits following acquired brain injury: A systematic review and meta-analysis. *NeuroRehabilitation, 36*(3), 367-377. doi:10.3233/NRE-151225

Wang, C., Yu, J. T., Wang, H. F., Tan, C. C., Meng, X. F., & Tan, L. (2014). Non-pharmacological interventions for patients with mild cognitive impairment: a meta-analysis of randomized controlled trials of cognition-based and exercise interventions. *Journal of Alzheimer's Disease, 42*(2), 663-678. doi:10.3233/JAD-140660

Wang, P., Liu, H. H., Zhu, X. T., Meng, T., Li, H. J., & Zuo, X. N. (2016). Action video game training for healthy adults: a meta-analytic study. *Frontiers in Psychology, 7*, 907. doi:10.3389/fpsyg.2016.00907

Weicker, J., Villringer, A., & Thone-Otto, A. (2016). Can impaired working memory functioning be improved by training? A meta-analysis with a special focus on brain injured patients. *Neuropsychology, 30*(2), 190-212. doi:10.1037/neu0000227

Woods, B., Aguirre, E., Spector, A. E., & Orrell, M. (2012). Cognitive stimulation to improve cognitive functioning in people with dementia. *Cochrane Database of Systematic Reviews*(2), CD005562. doi:10.1002/14651858.CD005562.pub2

Yang, H. L., Chan, P. T., Chang, P. C., Chiu, H. L., Sheen Hsiao, S. T., Chu, H., & Chou, K. R. (2018). Memory-focused interventions for people with cognitive disorders: A systematic review and meta-analysis of randomized controlled studies. *International Journal of Nursing Studies, 78*, 44-51. doi:10.1016/j.ijnurstu.2017.08.005

Supplementary Material 5. AMSTAR ratings

Author	Year	Item 1	Item 2	Item 3	Item 4	Item 5	Item 6	Item 7	Item 8	Item 9	Item 10	Item 11	Item 12	Item 13	Item 14	Item 15	Item 16
Alves	2013	Yes	No	Yes	Partial Yes	Yes	Yes	No	Partial Yes	Yes	Yes	No	Yes	Yes	Yes	Yes	Yes
Bahar-Fuchs	2019	Yes	Yes	Yes	Yes	Yes	No	Yes	Yes	Yes	No	Yes	Yes	Yes	Yes	Yes	Yes
Bhome	2018	Yes	Partial yes	No	Partial Yes	No	Yes	No	Partial Yes	Partial Yes	No	No	No	Yes	Yes	Yes	Yes
Chandler	2016	Yes	No	Yes	Partial Yes	Yes	Yes	No	Yes	No	No	No	No	No	Yes	Yes	Yes
Chiu	2017	Yes	No	No	Partial Yes	No	Yes	No	Partial Yes	Yes	No	Yes	No	No	No	Yes	Yes
Cooper	2012	Yes	No	No	Partial Yes	No	No	No	Yes	Partial Yes	No	No	Yes	Yes	No	Yes	Yes
das Nair	2016	Yes	Yes	No	Yes	Yes	Yes	Yes	Yes	Yes	No	Yes	No	Yes	Yes	No	Yes
Floyd	1997	Yes	No	Yes	Partial Yes	No	Yes	No	No	No	No	No	No	No	Yes	Yes	No
Folkerts	2017	Yes	No	Yes	Partial Yes	Yes	Yes	No	Yes	Yes	No	Yes	No	No	Yes	Yes	Yes
Gates	2019a	Yes	Yes	No	Yes	Yes	Yes	Yes	Partial Yes	Yes	Yes	Yes	No	Yes	Yes	No	Yes
Gates	2019b	Yes	Yes	No	Yes	Yes	Yes	Yes	Partial Yes	Yes	Yes	Yes	No	Yes	Yes	No	Yes
Gross	2012	Yes	No	No	Partial Yes	Yes	Yes	No	No	No	No	No	No	No	Yes	Yes	Yes
Hill	2017	Yes	Yes	Yes	Partial Yes	Yes	No	No	Partial Yes	Yes	No	Yes	Yes	Yes	Yes	Yes	Yes
Hindin	2012	Yes	No	No	Partial Yes	No	No	No	No	No	No	No	No	No	Yes	No	Yes
Hoefler	2016	Yes	No	No	Partial Yes	No	No	No	No	No	No	No	No	No	Yes	No	No
Hudes	2019	Yes	Yes	Yes	Partial Yes	Yes	No	No	No	Partial Yes	No	Yes	No	Yes	No	No	No
Huntley	2015	Yes	No	No	Partial Yes	Yes	Yes	No	Yes	Yes	No	Yes	Yes	Yes	Yes	Yes	Yes
Karr	2014	Yes	No	No	Partial Yes	No	Yes	Yes	No	No	No	No	Yes	Yes	Yes	Yes	Yes
Kelly	2014	Yes	Yes	No	Partial Yes	Yes	Yes	Yes	Partial Yes	Yes	No	Yes	No	No	No	No	Yes
Kim	2017	Yes	No	No	Partial Yes	Yes	No	Yes	Partial Yes	Yes	No	No	No	Yes	No	No	Yes
Kurz	2011	Yes	No	No	Partial Yes	Yes	No	No	Partial Yes	No	No	No	No	No	Yes	Yes	Yes
Lampit	2014	Yes	Yes	Yes	Partial Yes	Yes	No	No	Yes	Yes	No	Yes	Yes	Yes	Yes	Yes	Yes
Lawrence	2017	Yes	No	Yes	Partial Yes	Yes	No	No	No	No	No	Yes	No	No	Yes	Yes	Yes
Lee	2019	No	No	Yes	Partial Yes	No	No	No	Partial Yes	Yes	No	No	No	No	No	Yes	Yes
Leung	2015	Yes	Yes	No	Partial Yes	Yes	No	No	Partial Yes	Yes	No	Yes	No	Yes	Yes	yes	Yes
Leung	2017	Yes	No	Yes	Partial Yes	No	Yes	No	Partial yes	Yes	No	Yes	No	Yes	Yes	No	Yes
Loetscher	2013	Yes	Yes	Yes	Yes	Yes	Yes	Yes	Yes	Yes	No	Yes	No	Yes	Yes	No	Yes
Martin	2011	Yes	Yes	No	Partial Yes	Yes	No	Yes	Partial Yes	No	No	No	No	No	No	No	Yes
Melby-Lervag	2016	Yes	No	No	No	No	No	No	No	No	No	No	No	No	No	No	No
Metternich	2010	Yes	No	No	Partial Yes	Yes	Yes	No	Partial Yes	No	No	No	No	No	Yes	No	Yes
Mewborn	2017	Yes	Yes	No	Partial Yes	No	No	No	Partial Yes	Yes	No	Yes	Yes	Yes	Yes	Yes	No
Papp	2009	Yes	No	Yes	Partial Yes	No	No	No	Yes	No	No	No	No	No	No	No	No
Pinquart	2001	No	No	No	Partial Yes	No	No	No	No	No	No	No	No	No	Yes	No	No
Rogers	2018	Yes	Yes	Yes	Partial Yes	Yes	Yes	No	Yes	Partial Yes	No	Yes	Yes	Yes	Yes	Yes	Yes
Shao	2015	No	No	No	Partial Yes	Yes	Yes	No	Partial Yes	Yes	No	No	No	No	Yes	No	Yes
Sherman	2017	Yes	No	No	Partial Yes	Yes	No	No	Partial Yes	Yes	No	Yes	No	No	Yes	Yes	Yes
Smart	2017	Yes	No	No	Partial Yes	Yes	Yes	Yes	No	No	No	No	No	Yes	No	Yes	Yes
Song	2016	No	No	No	Partial Yes	No	No	No	No	no	No	No	No	No	No	Yes	Yes
Tetlow	2017	No	Yes	No	Partial Yes	No	No	No	No	Partial Yes	No	Yes	No	No	Yes	No	No
Toril	2014	Yes	No	No	Partial Yes	No	Yes	No	Partial Yes	No	No	No	No	No	Yes	Yes	Yes
Wang	2014	Yes	No	No	Partial Yes	No	Yes	No	Partial Yes	No	No	Yes	Yes	Yes	Yes	No	Yes
Wang	2016	Yes	No	No	Partial Yes	Yes	Yes	No	No	No	No	No	Yes	Yes	Yes	Yes	Yes
Weicker	2016	Yes	No	Yes	Partial Yes	Yes	No	No	No	No	No	No	No	No	Yes	Yes	Yes
Virk	2015	Yes	No	Yes	Partial Yes	Yes	Yes	No	Partial Yes	Yes	No	Yes	Yes	Yes	Yes	Yes	Yes
Woods	2012	Yes	Yes	No	Partial Yes	Yes	No	Yes	Yes	Yes	No	Yes	No	Yes	No	Yes	Yes
Yang	2018	No	No	No	Partial Yes	Yes	No	No	Partial	Yes	No	Yes	No	No	Yes	Yes	No

Supplementary Material 6. Sensitivity analysis including only reviews with ‘low’ or ‘moderate’ confidence rating

Population	Intervention	Outcome	Mean effect size estimate	No. of reviews
Healthy older adults	Cognitive training	Objective cognition	0.33 (0.00 to 0.65)	5
Mild cognitive impairment	Cognitive training	Objective cognition	0.37 (0.06 to 0.67)	3
Dementia	Cognitive stimulation	Objective cognition	0.37 (0.14 to 0.60)	3
Dementia	Cognitive training	Objective cognition	0.43 (-0.07 to 0.93)	5
